# Three Selected Edible Crops of the Genus *Momordica* as Potential Sources of Phytochemicals: Biochemical, Nutritional, and Medicinal Values

**DOI:** 10.3389/fphar.2021.625546

**Published:** 2021-05-13

**Authors:** Mashudu Muronga, Cristina Quispe, Phumudzo P. Tshikhudo, Titus A. M Msagati, Fhatuwani N. Mudau, Miquel Martorell, Bahare Salehi, Ahmad Faizal Abdull Razis, Usman Sunusi, Ramla Muhammad Kamal, Javad Sharifi-Rad

**Affiliations:** ^1^ Department of Agriculture and Animal Health, College of Agriculture and Environmental Sciences, University of South Africa, Florida, South Africa; ^2^ Facultad de Ciencias De La Salud, Universidad Arturo Prat, Iquique, Chile; ^3^ Pest Risk Analysis, Directorate Plant Health, Department of Agriculture, Land Reform and Rural Development, Pretoria, South Africa; ^4^ Nanotechnology and Water Sustainability Unit, College of Science Engineering and Technology, University of South Africa, Science Campus, Florida, South Africa; ^5^ School of Agriculture, Earth and Environmental Sciences, University of KwaZulu-Natal, Pietermaritzburg, South Africa; ^6^ Department of Nutrition and Dietetics, Faculty of Pharmacy, and Centre for Healthy Living, University of Concepción, Concepción, Chile; ^7^ Universidad de Concepción, Unidad de Desarrollo Tecnológico, UDT, Concepción, Chile; ^8^ Medical Ethics and Law Research Center, Shahid Beheshti University of Medical Sciences, Tehran, Iran; ^9^ Department of Food Science, Faculty of Food Science and Technology, Universiti Putra Malaysia, Selangor, Malaysia; ^10^ Natural Medicines and Products Research Laboratory, Institute of Bioscience, Universiti Putra Malaysia, Selangor, Malaysia; ^11^ Department of Biochemistry, Bayero University Kano P M B, Kano, Nigeria; ^12^ Department of Pharmacology, Federal University Dutse, Dutse, Nigeria; ^13^ Phytochemistry Research Center, Shahid Beheshti University of Medical Sciences, Tehran, Iran; ^14^ Facultad de Medicina, Universidad Del Azuay, Cuenca, Ecuador

**Keywords:** momordica, anti-microbial, photochemical, secondary metabolites, inter alia

## Abstract

*Momordica* species (Family Cucurbitaceae) are cultivated throughout the world for their edible fruits, leaves, shoots and seeds. Among the species of the genus *Momordica,* there are three selected species that are used as vegetable, and for medicinal purposes, *Momordica charantia* L (Bitter melon), *Momordica foetida* Schumach (Bitter cucumber) and *Momordica balsamina* L (African pumpkin). The fruits and leaves of these *Momordica* species are rich in primary and secondary metabolites such as proteins, fibers, minerals (calcium, iron, magnesium, zinc), *β*-carotene, foliate, ascorbic acid, among others. The extracts from *Momordica* species are used for the treatment of a variety of diseases and ailments in traditional medicine. *Momordica* species extracts are reputed to possess anti-diabetic, anti-microbial, anthelmintic bioactivity, abortifacient, anti-bacterial, anti-viral, and play chemo-preventive functions. In this review we summarize the biochemical, nutritional, and medicinal values of three *Momordica* species (*M. charantia*, *M. foetida* and *M. balsamina*) as promising and innovative sources of natural bioactive compounds for future pharmaceutical usage.

## Introduction

*Momordica charantia* L. (Bitter melon), *Momordica foetida* Schumach. (Bitter cucumber), *Momordica balsamina* L. (African pumpkin) are widely cultivated and they belong to the family Cucurbitaceae (Daniel et al., 2014). In some African countries; fruits, leafy shoots and the ripe seeds from these three species are harvested for consumption as vegetable, spices and leaves are mixed with water and consumed as beverages (Cantwell et al., 1996). *Momordica* species (spp.) are annual crops but can be regarded as perennial crop due to their performance during the season (Krawinkel and Keding, 2006; Tan et al., 2008; Costa et al., 2010; Chen et al., 2011; Abegunde et al., 2018). These crop species are mostly adapted to areas of minimum annual average rainfall such as those that receive an average of 400 mm as well as those with mild and frost-free winters (Tokhtar and Doan, 2014). *Momordica* spp. require a minimum temperature of 18°C during emergence and germination and during vegetative stage and flowering and an optimal temperature ranging between 24 and 27°C (Tan et al., 2008). Some researchers have stated that the crops adapt and perform well in deep and well-drained sandy loam and slit loam soils which are rich in organic matter and adapt well in soils with optimum soil pH ranging from 4.3 to 8.0 (Tan C. et al., 2015). However, they can adapt to alkaline soils with up to pH of 8.0 (Grover and Yadav, 2004; Harinantenaina et al., 2006; Tan et al., 2008). *Momordica* spp. are open-pollinated and consist of separate male and female flowers on the same plant (Palada and Chang, 2003). Flowering of *Momordica* species begins a month after planting until reaching dormancy. The fruits are orange in color and soft when ripe with black seeds (Bakare et al., 2010). The local South African indigenous traditional people have been using *Momordica* spp. as culinary species or enhance bitterness of the meat and of vegetables (Madala et al., 2014). However, studies on the trend of food consumption for rural residents in developing countries have often focused relatively on the economic factors such as prices, income, and market development Jia et al. (2017), and have rarely covered social and psychological influences.

Normally, *Momordica* spp. are consumed as vegetables and as nutrient supplements in Asia, Africa and South America and they are also used as medicinal herbs (Tan et al., 2008; Madala et al., 2014; Jia et al., 2017; Islam and Jalaluddin, 2019). Different parts of *Momordica* spp. are rich in both primary and secondary metabolites. The primary metabolites that are derived from *Momordica* spp. include sugars, proteins and chlorophyll (Hassan and Umar, 2006; Daniel et al., 2014), while secondary metabolites are alkaloids, flavonoids, tannins (Roškar and Lušin, 2012; Mada et al., 2013; Madala et al., 2016; Mostafa et al., 2018). *Momordica* spp. are good a source of phenolic compounds, gallic acid, gentisic acid (2,5-dihydroxyl benzoic acid), catechins, chlorogenic acid and epicatechin (Abegunde et al., 2018).

Generally, extracts from *Momordica* spp. are useful for the treatment of various diseases and many other ailments due to the several biological activities exerted such as anti-diabetic Tan et al. (2008), Madala et al. (2014), Jia et al. (2017), Islam and Jalaluddin (2019), Perera et al. (2021), Venugopal and Dhanasekaran (2021), anti-oxidant Acquaviva et al. (2013), Ozusaglam and Karakoca (2013), Huang et al. (2020), Perumal et al. (2021), anti-microbial (Kumar et al. (2009), Patel et al. (2010), Gupta et al. (2011), anti-bacterial (Koneri et al. (2006), Orhan et al. (2010), Leelaprakash et al. (2011), Puškárová et al. (2017), Chen et al. (2019), Islam and Jalaluddin (2019), Khadijeh et al. (2019), anti-viral Orhan et al. (2010), Santhosh Kumar et al. (2020), anti-fungal Santos et al. (2012), anthelmintic Beloin et al. (2005), Jia et al. (2017), insecticidal Pari et al. (2020), antiglycation Chen et al. (2011), anti-inflammatory Chen et al. (2019), Khadijeh et al. (2019), Perera et al. (2021), Venugopal and Dhanasekaran (2021), anti-thrombotic Patil and Rathod (2020), anti-allergic Asna et al. (2020), hepatoprotective Ramalhete et al. (2011), estrogenic Badhwar et al. (2020), hypolipidemic effect Aeri and Raj (2020), anti-carcinogenic Kesari et al. (2020), anti-proliferative Yue et al. (2020), anti-mutagenic Rolnik and Olas (2020), and chemo-preventive activities (Gupta, 2020).

*Momordica* spp. have been reported to have inhibitory effects against diabetes, cancer, and cardiovascular diseases in animal and humans (Trakoon-osot et al., 2013; Fongod et al., 2014; Ingle et al., 2017; Jabeen and Khanum, 2017; Perera et al., 2021). Some studies shows that the bioactivity effectiveness in lowering of blood sugar (Huang et al., 2008; Rahmatullah et al., 2012; Balouiri et al., 2016; Anjamma and Bhavani, 2018), in controlling eye disorders and enhancing eyesight due to the presence of *β*-carotene (Ludidi et al., 2019). In addition, some studies have suggested that *Momordica* spp. are used to treat gastrointestinal parasitic disease (Grover and Yadav, 2004), diarrhea, bleeding of gums Abidemi (2013), piles and hemorrhoids Lii et al. (2009), respiratory problems Leung et al. (2009) and skin infections (Kumar and Bhowmik, 2010). The anti-viral and anti-leukemic activities of extract from *M. balsamina* against liver cancer, leukemia, melanoma and solid sarcomas have also been reported (Lu et al., 2011; Aparicio et al., 2021).

*Momordica* spp. have flavonoids with anti-microbial activities (Orhan et al., 2010) and *Momordica* leaves extracts have shown anti-bacterial activities against *Staphylococcus aureus, Salmonella typhii, Escherichia coli, Klebsiella pneumoniae, Bacillus subtilis, Pseudomonas* sp. and *Entamoeba histolytica* (Tansey et al., 2004; Froelich et al., 2007; Ghosh, 2014; Balouiri et al., 2016) and the fruit extract possesses anti-microbial activity against *Helicobacter pylori* (Mwambete, 2009).

The use of the plant extracts of *Momordica* spp*.* to fully explored their effectiveness, may lead to new drug discovery or advance the use of indigenous herbal medicines for orthodox treatment of certain diseases (Rahmatullah et al., 2012). The aim of this review was to discuss the biochemical, nutritional, and medicinal values of three *Momordica* spp. (*M. charantia*, *M. foetida* and *M. balsamina*) as promising and innovative sources of natural bioactive compounds for future pharmaceutical usage.

## Methodology

### Type of Review

The systematic review method involved six steps: 1) scoping; 2) planning; 3) search process and identification of articles; 4) Screening of articles; 5) assessment of relevancy and 6) Presentation and interpretation of the results. As a rigorous and unbiased method of evaluating and screening the literature attributed to its replicability and exhaustiveness, it is a method of choice suitable for summarizing the current literature on the three *Momordica* spp (*M. charantia*, *M. foetida* and *M. balsamina*).

### Literature Sources and Searching Procedure

**FIGURE 1:**
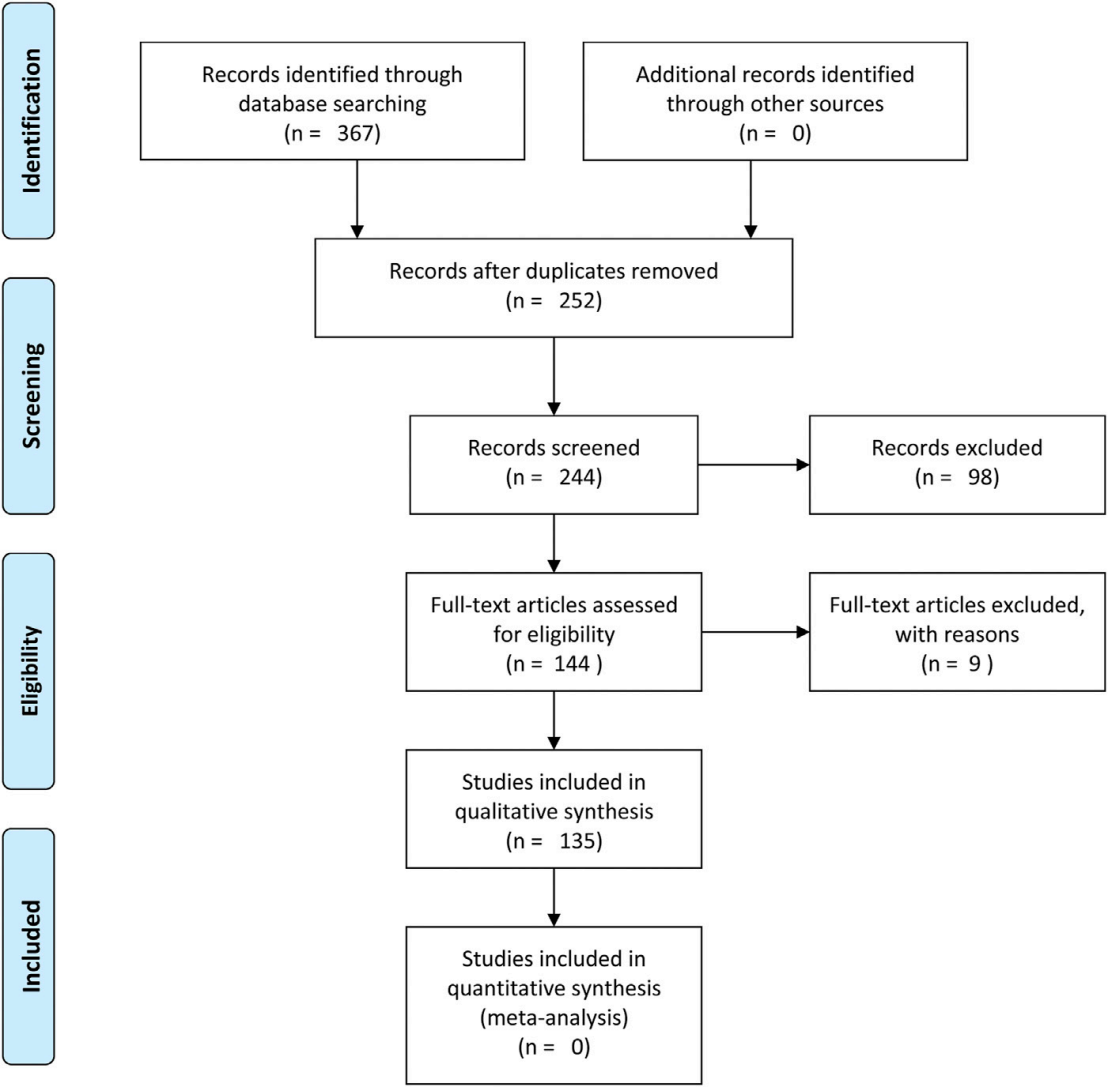
PRISMA flow diagram.

The search was conducted from six electronic database (Web of Science, Science.gov, Science Direct, Google Scholar, Scopus and Ebscohost) using the following guidelines: 1) “Momordica charantia” OR “Bitter melon”, 2) “Momordica. Foetida” or “Bitter cucumber”, and 3) “Momordica. Balsamina” OR “African pumpkin”. The common search for peer reviewed article used included the biochemical, nutritional, and medicinal values or composition of *Momordica* spp. The same search was also conducted using the common names of the species. The search was filtered based on the year of publications (Year = 1990–2020) to obtain most recent literature however, it did not exclude the old publications (Year<1990) which form the basic knowledge of the associated literature. Since, the study was limited to qualitative synthesis, the PRISMA 2009 Flow Diagram (Moher et al., 2009) was to modified to exclude the meta-analysis aspects (Figure 1).

## Biochemical Composition and Activity of the Three Selected *Momordica* Species

### *Momordica charantia* L.

*M. charantia* is a good source of primary metabolites such as carbohydrates, fibers, proteins, minerals, and vitamins. The most important chemical constituents from *M. charantia* include heteropolysaccharides (e.g., arabinose, galactose, glucose, mannose and rhamnose); proteins and peptides (e.g., momordins, momorcharins, etc); terpenoids and saponins (e.g., cucurbitanes and cucurbitacines); flavonoids and phenolic compounds (Chen et al., 2003; Harinantenaina et al., 2006; Schrot et al., 2015; Dandawate et al., 2016; Lee et al., 2021). *M. charantia* also contains triterpenes, proteins, steroids, alkaloids, lipids (lauric, myriaatic, palmitic, stearic, linoleic, and eleostearic acids), phenolic compounds and minerals (Cu, Fe, Mg, Zn, and Ca) (Yuwai et al., 1991; Kumar and Bhowmik, 2010; Joseph and Jini, 2013; Tokhtar and Doan, 2014; Tan E. S. et al., 2015; Yoshime et al., 2016). The leaves and flowers of *M. charantia* contains triterpenoids (momordicine and charantin), carotenoids (antheraxanthin, lutein, violaxanthin, *α*-carotene, and *β*-carotene), and phenylpropanoids (caffeic acid, chlorogenic acid, epicatechin, gallic acid, p-coumaric acid, rutin, and *trans*-cinnamic acid) (Sathasivam et al., 2021). The water content of the fruits of *M. charantia* is about 93.2%, while protein and lipids content ranges between 18.02 and 0.76% (Saad et al., 2017). The fruit pulp of *M. charantia* consists of soluble pectin and galactouronic acid. Moreover, the fruits consist of glycosides, saponins, alkaloids, reducing sugars, resins, phenolic constituents, fixed oil and free acids (Bakare et al., 2010). Green fruits of *M. charantia* contain Vitamin A, Vitamin C, and Vitamin *p*, thiamine, riboflavin, niacin, and minerals (Gupta et al., 2011). The essential oil, obtained from water stressed seeds also contains sesquiterpenes, phenylpropanoids and monoterpenes, however seed oil contain tocopherols and polyphenols (Nyam et al., 2009). The phenolic compounds that are found on all parts of the crop protect the plant from oxidative damage (Yoshime et al., 2016; Wardani et al., 2021).

Among the bioactive compounds that have been isolated from the fruits of *M. charantia* and elucidated by nuclear magnetic resonance (NMR) and mass spectrometry (MS) spectroscopic techniques include Monordicophenoide A (4-hydroxyl-benzoic acid 4-*O*-*β*-D-apiofuranosyl (1→2)-*O*-*β*-D-glucopyranoside; dihydrophaseic acid 3-*O*-*β*-D-glucopyranoside; momordicolide (10E)-3-hydroxyl-dodeca-10-en-9-olide; 6,9-dihydroxy-megastigman-4,7-dien-3-one (blumenol); adenosine; guanosine; uracil; and cytosine (Li et al., 2009). Those isolated from the leaves of *M. charantia* include Momordicin I, Momordicin IV, aglycone of Momordicoside, aglycone of Momordicoside L; and Karavilagenin D were identified (Li et al., 2015). The essential oil such as trans-nerolidol, apiole, cis-dihydrocarveol, and germacrene D were isolated from the seeds of *M. charantia* and identified by gas chromatography mass spectrometry (GC/MS) (Braca et al., 2008).

Main phenolic acids found in *M. charantia* are gallic acid, chlorogenic acid, catechin, caffeic acid, p-coumaric acid, and ferulic acid (Lee et al., 2017). Compounds such as capxanthin, lutein, zeaxanthin, *β*-cryptoxanthin, lycopene, *α*-carotene, and *β*-carotene have also detected from the fruit of *M. charantia* (Lee et al., 2017). The variation in phenolic acids and carotenoids contents at different maturity stages create a strong relationship with the anti-oxidant activities of *M. charantia* fruit. The crop consists of non-essential amino acids such as arginine, alanine, aspartic acid, glycine, glutamic acid, proline, histidine and serine. Such non-essential amino acids are inherently higher in concentration, as compared to essential amino acids such as leucine, cysteine, phenylalanine, isoleucine, methionine, lysine, threonine, tyrosine and valine. From the leaves of *M. charantia* leucine and aromatic (tyrosine and phenylalanine) were the predominant amino acids, while aspartic acid and glutamic acid are major non-essential amino acids of this plant (Khanna, 2004).

Other reported phytochemical compounds from *M. charantia* plant were obtained after being extracted and their chemical structures identified using NMR. These include *β*-sitosterol; (23E)-5β,19-epoxycucurbita-6,23-diene-3β,25-diol; daucosterol; uracil; and allantoin using spectroscopic analysis including MS and 1H- and C-NMR (Kim et al., 2018). The structures of momordicosides K were identified as 7-*O*-*β*-D-glucopyranosides of 3β,7β-dihydroxy-25-methoxy-cucurbita-5; 23-dien-19-al; and momordicosides L as 3β,7β,25-trihydroxy-cucurbita-5,23-dien-19-al (Okabe et al., 1982).

### *Momordica foetida* Schumach

*M. foetida* have been reported to contain secondary metabolites such as sitosterylglucoside, 5,25-stigmastadien-3β-ylglucoside, and 1β-hydroxyfriedel-6-en-3-one, and a lot of cucurbitane-type triterpenoid derivatives (Molehin and Adefegha, 2014). Phenolic glycosides of *M. foetida* include eriodictyol-, kaempferol- 5,7,4′-trihydroxyflavanone, and 5,7-dihydroxychromone-7-*O*-*β*-D-glucopyranoside (Froelich et al., 2007).

The crop species of *M. foetida* contain 25-trihydroxycucurbita-5,23-dien-19-al and 3β,7β-dihydroxy-25 methoxycucurbita-5,23-dien-19-al; 3β, 7β, 23ξ-trihydroxycucurbita-5,24-dien-19-al, 3β, 7β. Others include 5β,19-epoxy-25-methoxycucurbita-6,23-diene-3β,19-diol,5β,19-epoxycucurbita-6,23-diene-3β,19,25-triol,5β,19-epoxy-19 methoxycucurbita-6, 23-diene-3β,25-diol,5β,19-epoxy-19,25-dimethoxycucurbita-6,23-dien-3β-ol and 5β,19-epoxy-25-methoxy-cucurbita-6,23-dien-3β-ol (Mulholland et al., 1997). Secondary metabolites detected in *M. foetida* are steroids, alkaloids, cardiac glycosides, phenolics, tannins, flavonoids and saponin (Ndam et al., 2014).

The chemical structures of the isolated compounds include 3β,7β-dihydroxyl-cucurbita-5,23,25-trien-19-al followed by Kaempferol-3-*O*-*β*-D-glucopyranoside have anti-microbial activity (Odeleye et al., 2009). The fruits and leaves of *M. foetida* crop contain Momordicines and Foetidin (identical to Charantin). Phytochemical analysis of *M. foetida* revealed phenolic glycosides such as eriodictyol-, 5,7,4′-trihydroxyfl avanone-, kaempferol- and 5,7-dihydroxychromone-7-*O*-*β*-D-glucopyranoside (Froelich et al., 2007). Other phytochemical analysis of *M. foetida* extract led to the presence of alkaloids, saponins and cardenolides (Odusote and Awaraka, 2004).

### *Momordica balsamina* L.

The leaves, fruits, seeds, and bark of the *M. balsamina* contains secondary metabolites such as alkaloids, flavonoids, glycosides, steroids, terpenes, cardiac glycoside, saponins, tannins and lectins (Talukdar and Hossain, 2014). The matured seeds are the main source of plant lectins (Abegunde et al., 2018). The primary metabolites include crude lipid (2.66%), common sugars, proteins (11.29%), crude fiber (29%), energy, amino acid, carbohydrates (39%) and chlorophyll (Hassan and Umar, 2006; Thakur et al., 2009; Orhan et al., 2010; Ibrahim et al., 2014; Birla, 2016; Abegunde et al., 2018).

The leaves are an important source of nutrients, in addition they contain 17 amino acids and an adequate mineral composition (Hassan and Umar, 2006; Talukdar and Hossain, 2014; Wardhani et al., 2015; Wang et al., 2016; Nitu and Patidar, 2017). *M. balsamina* is rich potassium, calcium, magnesium, sodium, phosphorus, manganese, zinc, and iron which contribute to prevent micronutrient deficiencies in human.

## Nutritional Value of the Three Selected *Momordica* Species

### *Momordica charantia* L.

*M. charantia* fruit is commonly eaten unripe as a vegetable and is also used as herbal plant that is useful in metabolic and physiological processes of the human body. It is advisable to add peeled fruits to minimize bitter taste before preparation which involves soaking the fruits and shoots and then boiled, fried or pickled (Kumar and Bhowmik, 2010). *M. charantia* fruits are known to have high levels of nutritional value (protein, vitamins, thiamine, riboflavin, calcium and iron) compared to other cucurbits it has higher in folate and Vitamin C and the vine tips as an excellent source of Vitamin A. *M. charantia* leaves can be harvested, cooked and consumed alone or mixed with other indigenous vegetables to limit the bitterness (Hassan and Umar, 2006). The leaves and fruits of *M. charantia* have been used to make teas and beer or season soups in the Western world (Kumar and Bhowmik, 2010).

*M. charantia* contains iron, *β*-carotene, calcium potassium, vitamins, phosphorus and good dietary fiber (Daniel et al., 2014). About 93.2% of the fruit of *M. charantia* is composed of water, while protein and lipids account for 18.02 and 0.76%, respectively, (Li et al., 2009). Leaves powder from *M. charantia* contained 38% water, 38.2% dietary fiber, 67% minerals, 45% crude protein and 27% crude fat (Anilakumar et al., 2015). Protein lectin that is found on the seed of *M. charantia* is responsible for the inhibition of protein synthesis in the intestinal walls of an animal without producing any gastrointestinal symptoms in humans.

*M. charantia* seeds may induce fauvism in humans with glucose-6 phosphate dehydrogenase deficiency (Harinantenaina et al., 2006; Krawinkel and Keding, 2006; Ludidi et al., 2019). Some studies reported that *M. charantia* leaf powder inhibited adipocyte hypertrophy in diet-induced obese rats due to the process of decreasing lipogenic genes including fatty acid synthase, acetyl CoA carboxylase, lipoprotein lipase and adipocyte fatty acid and protein in epididymis fat (Tansey et al., 2004; Nerurkar et al., 2010; Anilakumar et al., 2015).

*M. charantia* reduced the accumulation of visceral fat that was fed to rats with high fat diet it and thus help in reducing insulin sensitizing and glucose due to its anti-adiposity effect (Chen et al., 2003; Huang et al., 2008; Dellavalle et al., 2011). Circulating levels of catecholamine and non-esterified fatty acids, lipid oxidation in the liver and muscle are reported to be as a result of *M. charantia* supplemented rat (Cohen et al., 1998; Waako et al., 2005; Harinantenaina et al., 2006; Bulbul, 2016; Devyani et al., 2016).

### *Momordica foetida* Schumach

The crop is commercially grown for their nutritional value and they are also used for medicinal purposes (Waako et al., 2005). The leaves of *M. foetida* are harvested from the wild and consumed when they are prepared as a vegetable in Gabon, Sudan, Uganda, Tanzania, South Africa and Malawi and some other African countries. The leaves of *M. foetida* are harvested, boiled and consumed with beans or peas together with a staple food. It is utilized in small amounts as a famine food and in emergency situations.

Traditional people prefer to consume *M. foetida* pulp of ripe fruits even if it has bitter taste (Mada et al., 2013; Mostafa et al., 2018; Ludidi et al., 2019). *M. foetida* leaves can be mixed with other vegetables to make a stew. It is also sauced with shrimp, meat, pork, and chicken and served with gravy. The leaves are mixed with ground peanuts and honey as a sauce in meat (Thakur et al., 2009; Dellavalle et al., 2011; Oronje et al., 2012; Sen and Batra, 2012; Fongod et al., 2014; Talukdar and Hossain, 2014; Bulbul, 2016; Poyraz and Derdovski, 2016; Nitu and Patidar, 2017; Mostafa et al., 2018).

*M. foetida* leaves are used as fodder and protein supplement in some countries and are suitable for increasing rabbits weight (Jabeen and Khanum, 2017). However, reports from Kenya state that cattle avoid *M. foetida* because of its unusual smell and bitter taste.

Nutritional composition of *M. foetida* leaf is energy, protein, fiber, calcium, iron, magnesium, zinc, *β*-carotene, foliate and ascorbic acid (Oloyede and Aluko, 2012; Puškárová et al., 2017; Abegunde et al., 2018). The leaves of *M. foetida* contain considerable amount of anti-oxidants and are capable of inhibiting lipid peroxidation (Oloyede and Aluko, 2012; Acquaviva et al., 2013; Anilakumar et al., 2015; Dzotam et al., 2016; Puškárová et al., 2017; Anjamma and Bhavani, 2018). Triterpenes are found in both *M charantia* and *M. foetida*, particularly in the fruits and seeds, and are potentially cytotoxic.

### *Momordica balsamina* L.

*M. balsamina* is important in producing nutrients supplements due its high protein and fat values with low fiber content (Bakare et al., 2010; Costa et al., 2010; Roškar and Lušin, 2012; Ghosh, 2014; Madala et al., 2014; Talukdar and Hossain, 2014; Abegunde et al., 2018). The leaves of *M. balsamina* are an important source of nutrients, including 17 amino acids (Cohen et al., 1998; Hassan and Umar, 2006; Costa et al., 2010; Semenya and Potgieter, 2015). *M. balsamina* leaves are mixed with peanuts and honey and used as a meat sauce (Dellavalle et al., 2011; Oronje et al., 2012; Sen and Batra, 2012; Fongod et al., 2014; Bulbul, 2016; Poyraz and Derdovski, 2016; Mostafa et al., 2018).

The fruit of *M. balsamina* contains the red, soft flesh that surround the edible seed (Tan C. et al., 2015; Birla, 2016; Devyani et al., 2016; Souda et al., 2018). The tender fruits and shoots of *M. balsamina* are usually boiled with meat and both can be decided to be added to the soup. Young fruits of *M. balsamina* are cooked and eaten, after peeling it is advisable to put on the salt water to minimize the bitterness before cooking, additionally the South Africans and Nigerians appreciate the fruit for its better taste (Acquaviva et al., 2013; Ghosh, 2014; Souda et al., 2018). The fruits of *M. balsamina* are frequent ingredients used in Indo Pakistan pickles and they are often used in curries and meat dishes.

Tender shoots of *M. balsamina* are usually utilized with Okra soup by the Kanuris of Borno State where the plant is locally known as the vegetable (Jabeen and Khanum, 2017). *M. balsamina* is preferred as an ingredient in aphrodisiac preparations however in Senegal they prefer to use the fruits as purgative agents, poultice and vermifuge (Leung et al., 2009; Souda et al., 2018).

## Medicinal Value of the Three *Momordica* Species

### *Momordica charantia* L.

*M. charantia* has medicinal value and is also used as a vegetable. The compounds present in *M. charantia* exert anti-diabetic, anti-cancerous and anti-tumorous, anti-microbial, anti-viral, anti-helmintic, antimalarial, anti-ulcerative and immunomodulatory activities (Gupta et al., 2011; Kulkarni et al., 2021; Lur et al., 2021; Malik et al., 2021). Some of the structures of compounds isolated from *M. charantia* are listed in Table 1.

**TABLE 1:** Compounds extracted from *Momordica charantia* L. and their health benefits.

Compound	Structure	Health benefits	References
Momordicoside F_2_	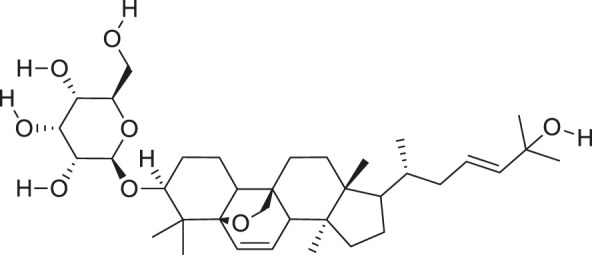	Anti-inflammatory and anti-cancer	Ju et al. (2004), Cao et al. (2018)
Goyaglycoside B	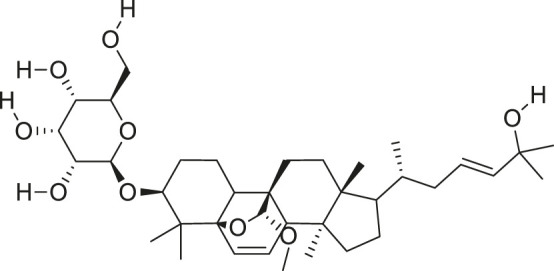	Anti-cancer	Raina et al. (2016), Cao et al. (2018)
Karaviloside III	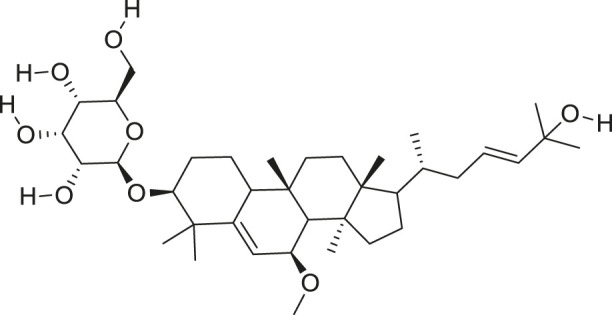	Anti-aging	Cao et al. (2018)
Charantoside VI	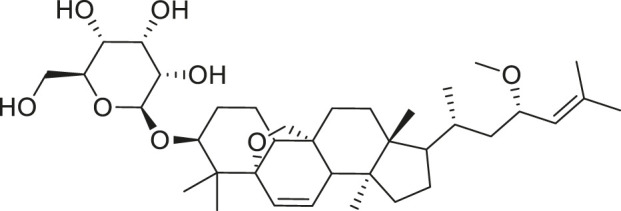	Anti-aging	Cao et al. (2018)
Charantagenin E	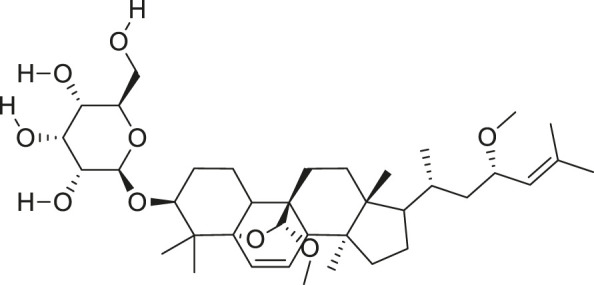	Anti-aging	Cao et al. (2018)
Charantoside II	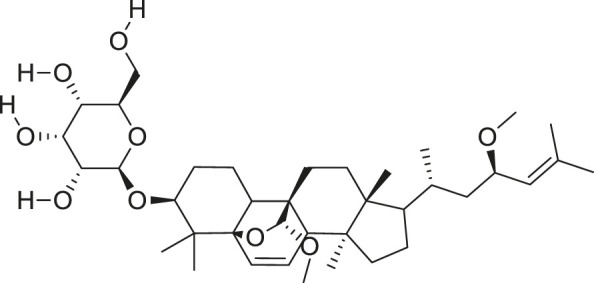	Anti-aging	Cao et al. (2018)
Momordicoside G	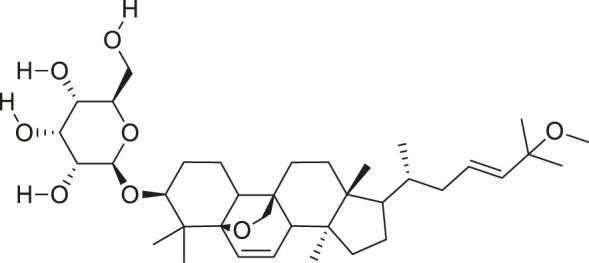	Anti-aging	Cao et al. (2018)
Goyaglycoside D	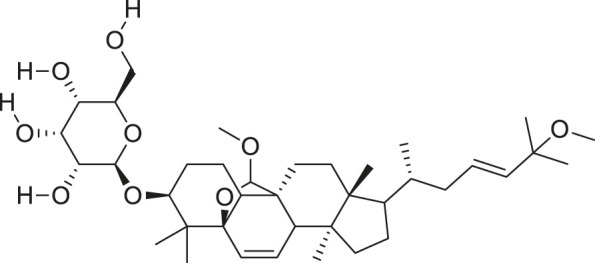	Anti-cancer	Wang et al. (2012), Cao et al. (2018)
Stigmasterol glucoside	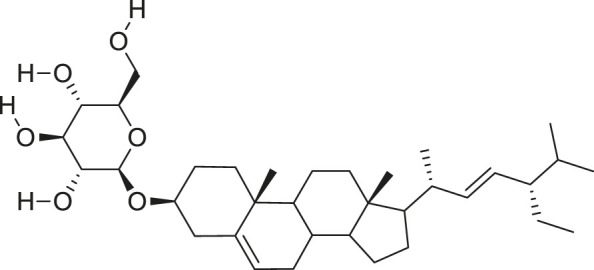	Anti-angiogenic and anti-cancer	Desai and Tatke, (2015), Kangsamaksin et al. (2017)
β-sitosterol glucoside	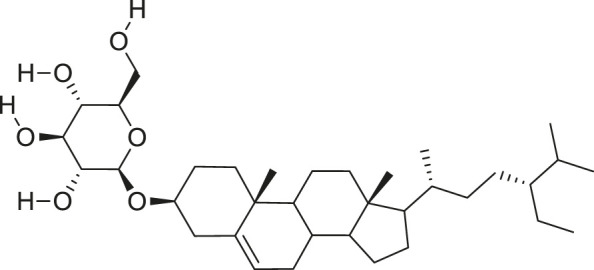	Cholesterol reduction and immune system modulation	Kaur et al. (2011), Desai and Tatke, (2015)
(19R)-7β,19-epoxy-19-methoxycucurbita-5,24-dien-3β,23-diol	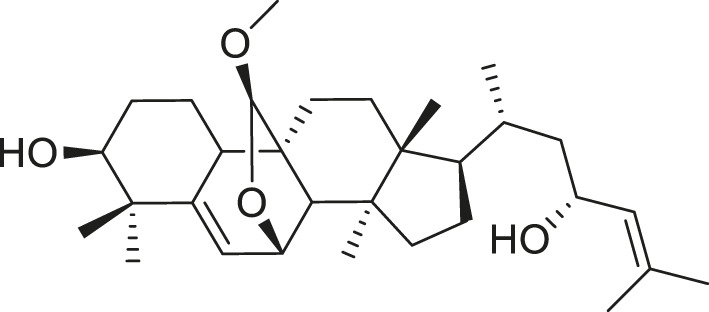	Anti-aging	Jiang et al. (2016), Cao et al. (2018)
Kuguacin J	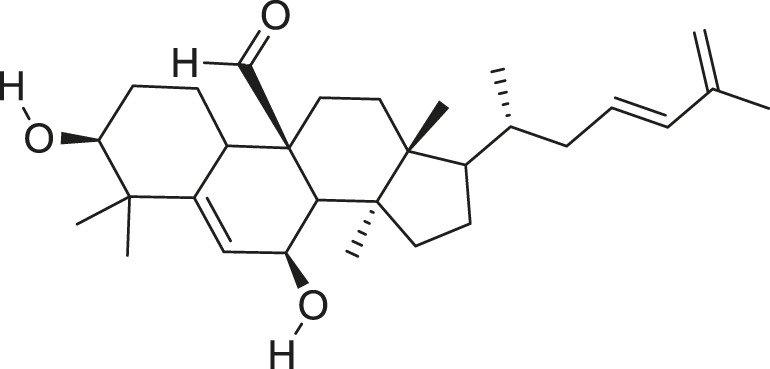	Anti-cancer	Limtrakul et al. (2013), Chen et al. (2015)
Cucurbitane triterpenoid	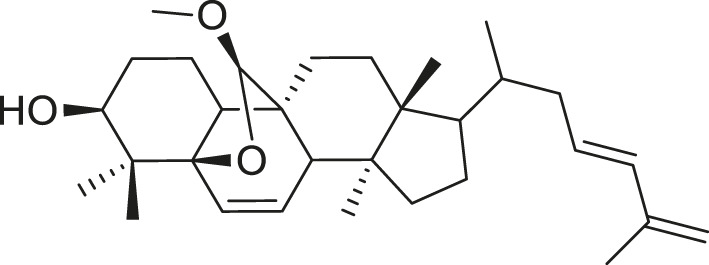	Prevention and therapy of human breast cancer	Liu et al. (2009), Bishayee et al. (2011)
Cucurbitane triterpenoid	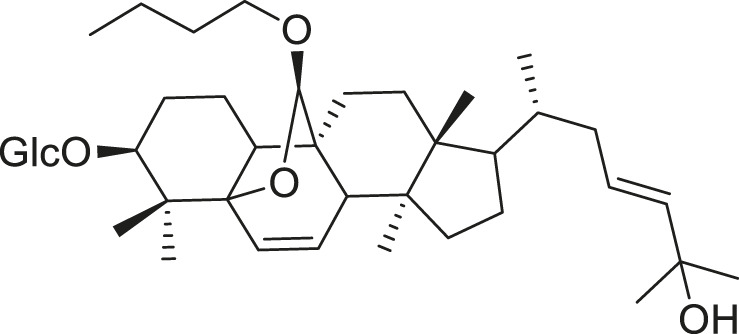	Anti-cancer	Liu et al. (2009), Bishayee et al. (2011)
Steroidal glycoside	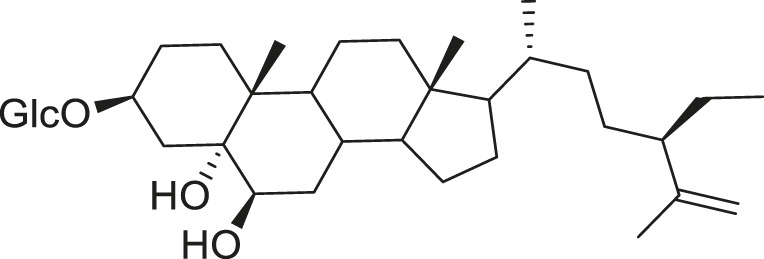	Reduce blood sugar level	Liu et al. (2009), Tan et al. (2015b), Lee et al. (2021)

Various medicinal properties have been claimed to be associated with *M. charantia* and they include anti-diabetic, abortifacient, anthelmintic, contraceptive, anti-malarial and laxative are used for various treatment such as dysmenorrhea, eczema, emmenagogue, galactagogue, gout, jaundice, kidney (stone), leprosy, leucorrhea, pneumonia, psoriasis, rheumatism, scabies, and piles (Grover and Yadav, 2004; Kumar and Bhowmik, 2010; Chen et al., 2011; Acquaviva et al., 2013; Ghosh, 2014; Bulbul, 2016; Perera et al., 2021).

The fruit juice and a leaf tea extracted from *M. charantia* can be used for the treatment of diabetes, malaria, colic, sores and wounds, infections, worms and parasites, as an emmenagogue, and for measles, hepatitis, and fevers (Kumar and Bhowmik, 2010). The anti-oxidants and chemo-protective are mostly available on *M. charantia* fruit extracts drug that helps with reducing risk of cancer (Dzotam et al., 2016; Khalid et al., 2021). The leaves of *M. charantia* are used for treatment of wounds as well against worms and parasites. *M. charantia* has been used as a traditional medicine in China, India, Africa, and the south-eastern United States (Grover and Yadav, 2004). In the 1980s, the seeds of *M. charantia* were investigated in China as a potential contraceptive (Kumar and Bhowmik, 2010).

Natural products from *M. charantia* have been reported to significantly decrease prostaglandin E2, interleukin and tumor necrosis factor and increases transforming growth factor and IL-10 secretion in RAW 264.7 macrophages, Caco-2 cells and THP-1 cells (Tan et al., 2008). The *M. charantia* fruit effectively enhanced T helper 2 hormonal responses and T helper 1 cellular immunity. A mixture of steroidal saponins contribute to the hypoglycemic and anti-hyperglycemic activity of *M. charantia* (Nitu and Patidar, 2017). Gentisic acid found as an active metabolite of salicylic acid and relation of the anti-inflammatory property of salicylic acid by inhibiting cyclooxygenase-2 (COX-2) mRNA expression and activity as well as prostaglandin E2 (Lii et al., 2009; Semenya and Potgieter, 2015; Wardhani et al., 2015; Madala et al., 2016; Wang et al., 2016).

*M. charantia* have been reported to exhibit anti-diabetic, anthelmintic, abortifacient, anti-bacterial, anti-viral, hypoglycemic agents and chemo-preventive functions (Grover and Yadav, 2004; Costa et al., 2010; Ghosh, 2014; Bulbul, 2016; Dzotam et al., 2016; Ludidi et al., 2019; Kulkarni ei al. 2021). The fruits and leaves of *M. charantia* display various biological activities including anti-diabetic, anti-rheumatic, and anti-ulcer, anti-inflammatory and anti-tumor (Dzotam et al., 2016). *M. charantia* contains various chemicals that have a hypoglycemic activity which reduces the amount of sugar in the blood, furthermore it stimulates appetite and helps in the entire digestion process. *M. charantia* is used for the treatment of digestive problems (Ludidi et al., 2019).

The leaves of *M. charantia* are used to treat diabetes, intestinal gas, promote menstruation, and as anti-viral agent against measles and hepatitis viruses (Tansey et al., 2004; Leung et al., 2009; Costa et al., 2010; Nerurkar et al., 2010; Nitu and Patidar, 2017; Perera et al., 2021; Liu et al., 2021). *M. charantia* is used by most of the culture for minimizing blood glucose and treating diabetes and as the other admired herbal resource (Leung et al., 2009; Birla, 2016). Hypoglycemic potential of *M. charantia* has been demonstrated in patients with type 2 diabetes. Emerging evidence also suggest that hypolipidemic action of *M. charantia* lowers serum cholesterol, hepatic total cholesterol and triglyceride and elevated cholesterol (Chen et al., 2003; Tan et al., 2008). The crop juice is believed significantly to reduce lipid accumulation and increase lipolysis in primary human adipocytes (Tan E. S. et al., 2015; Ingle et al., 2017). Animal studies indicate that *M. charantia* juice reduces body weight by inducing a reduced adipose hypertrophy, inhibition of lipogenic genes and enlarged plasma catecholamines (Nerurkar et al., 2010).

The ripe fruit of *M. charantia* fruits had been used as a remedy for tumors, asthma, skin infections and hypertension (Semenya and Potgieter, 2015). The seeds are surrounded by sweet red fleshy pulp that is edible tasting like watermelon (Poyraz and Derdovski, 2016). In some parts of West Africa, the leaves of *M. charantia* are cooked as part of green vegetable soup for post-natal mothers to prevent loss of blood during labor and purify breast milk. However, *M. charantia* can be recommended to use for livestock feeding as supplement for protein (Kuete et al., 2010).

### *Momordica foetida* Schumach

Traditional medicinal uses are numerous, and many are shared with other *Momordica* spp. In East and Central Africa, *M. foetida* extracts are used to treat hypertension, peptic ulcers, and diabetes. Foetidin isolated from *M. foetida* is capable of reducing blood-glucose level in fasting albino rats (Marquis et al., 1977). Likewise, *M. foetida* is used for treatment of digestive problems due to its hypoglycemic activity that reduces the amount of sugar in the blood and also stimulate appetite and helps in the entire digestion process (Leung et al., 2009; Kumar and Bhowmik, 2010; Chen et al., 2011; Daniel et al., 2014; Birla, 2016).

Additionally, *M. foeida* has slight anti-spasmodic and anti-cholinergic effects (Hassan and Umar, 2006; Acquaviva et al., 2013; Ingle et al., 2017; Puškárová et al., 2017). Some of the structures of compounds extracted from *M. foetida* are listed in Table 2.

**TABLE 2:** Compounds extracted from *Momordica foetida* Schumach. and their health benefits.

Compound	Structure	Health benefits	References
Quercetin malonl-glycoside	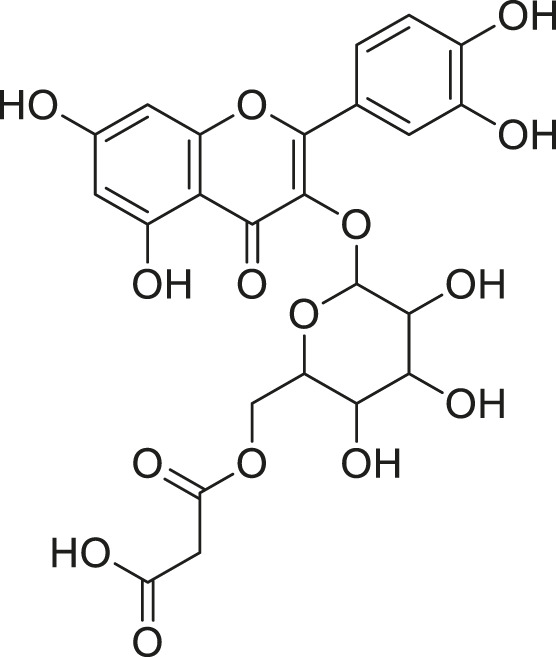	Lowers blood pressure and inflammation	Khoza et al. (2016), Dabeek and Marra, (2019)
Kaempferol malonyl-diglycoside	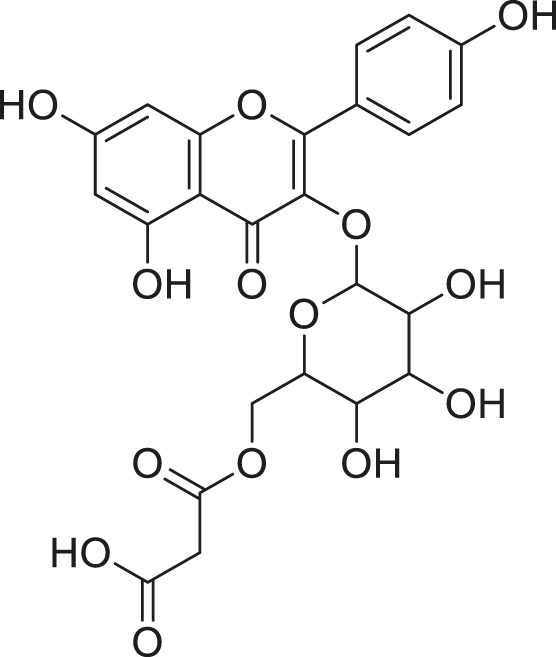	Anti-tumor, anti-oxidant and anti-inflammatory	Khoza et al. (2016), Abegunde et al. (2018)
Kaempferol Acetyl- glycoside	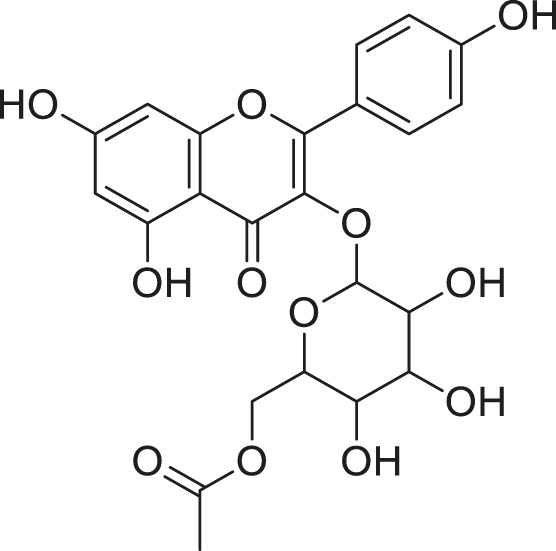	Anti-tumor, anti-oxidant and anti-inflammatory	Khoza et al. (2016), Abegunde et al. (2018)
Quercetin acetyl- glycoside	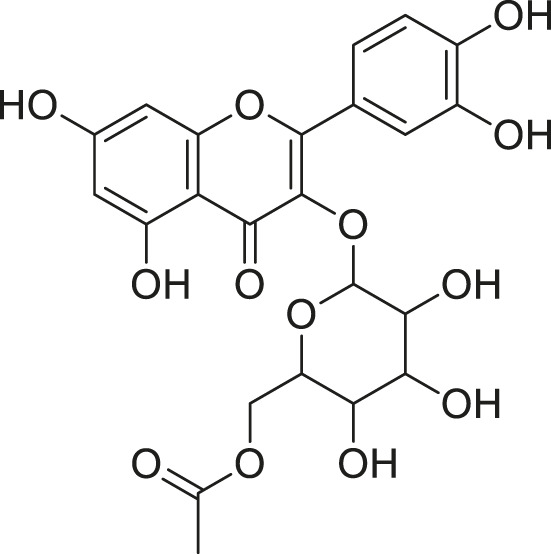	Lowers blood pressure and inflammation	Khoza et al. (2016), Dabeek and Marra, (2019)
Isorhamnetin acetyl-glycoside	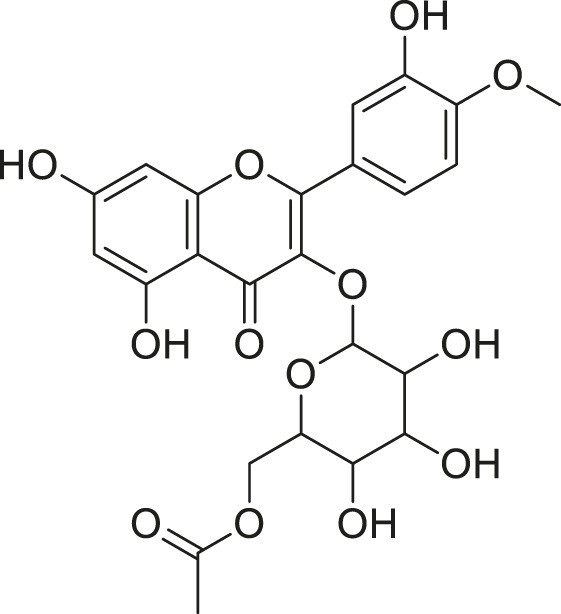	Anti-oxidant, anti-viral, anticancer, anti-microbial, and anti-inflammatory	Khoza et al. (2016), Kandakumar and Manju, (2017)
Isorhamnetin glycoside	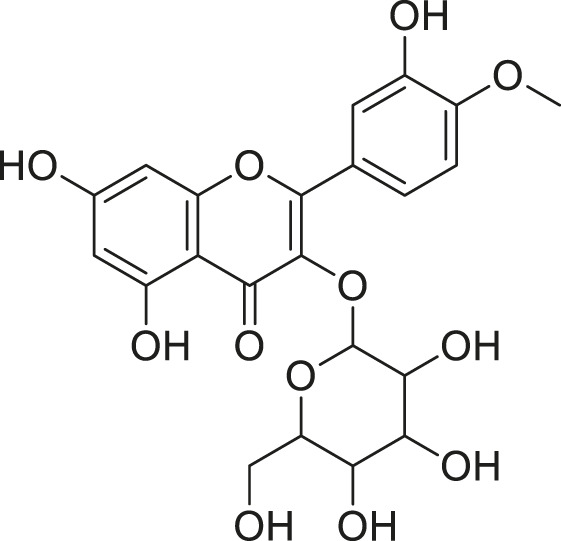	Anti-oxidant, anti-viral, anticancer, anti-microbial, and anti-inflammatory	Khoza et al. (2016), Kandakumar and Manju, (2017)
Quercetin glycoside	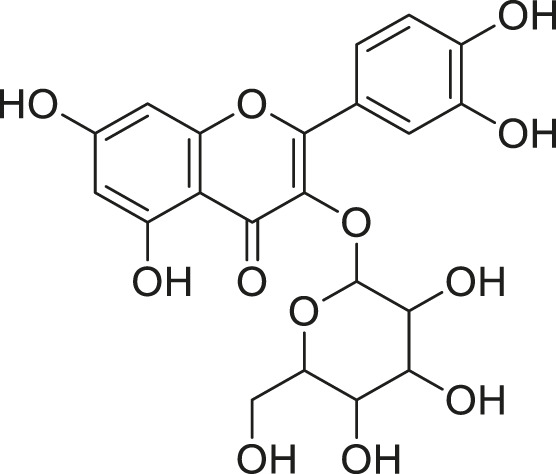	Lowers blood pressure and inflammation	Khoza et al., (2016), Dabeek and Marra, (2019)
Kaempferol glycoside	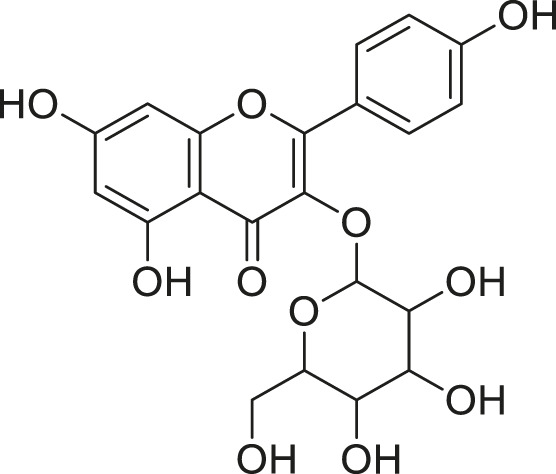	Anti-tumor, anti-oxidant and anti-inflammatory	Khoza et al. (2016), Abegunde et al. (2018)
Kaempferol diglycoside	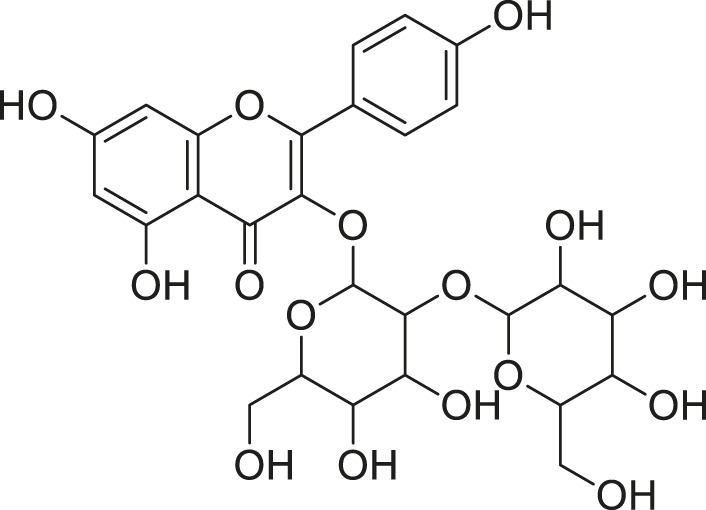	Anti-tumor, anti-oxidant and anti-inflammatory	Khoza et al. (2016), Abegunde et al. (2018)
Quercetin diglycoside	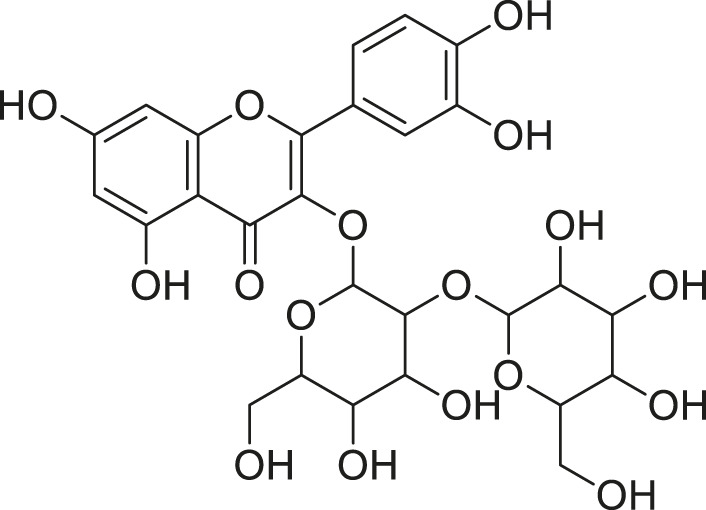	Lowers blood pressure and inflammation	Khoza et al. (2016), Dabeek and Marra, (2019)

Aqueous extract of *M. foetida* has anti-oxidant activity due to high content in phenolic and flavonoid compounds (Acquaviva et al., 2013). Oxidative stress is closely related with Alzheimer and Parkinson diseases, diabetes, rheumatoid arthritis, and several diseases. The use of anti-oxidants in pharmacology has been instrumental in treatments of stroke and neurodegenerative diseases (Jabeen and Khanum, 2017).

The juice of *M. foetida,* dried and crushed leaves are used to relieve cough, intestinal disorders, headache, earache, toothache and as an cure for snake bites (Jabeen and Khanum, 2017). Leaves can be dried or boiled and used to treat skin problems, spitting cobra poison and malaria. The roots of *M. foetida* may be stated to be poisonous, and the crushed seeds are used in East Africa to cure of constipation. *M. foetida* helps to reduce weight in obese people (Nerurkar et al., 2010; Talukdar and Hossain, 2014).

The fruit pulp of *M. foetida* is said to be poisonous to common pests such as weevils, moths and ants, making it to be utilized as potential insect repellent in Tanzania. Momordocin isolated from *M. foetida* has insecticidal ability (Olaniyi and Marquis, 1975; Pinar et al., 1983; Pirillo et al., 1995). In Uganda the *M. foetida* whole plant is use on their cattle as an ox pecker repellent. In Gabon, *M. foetida* soaked and dried leaves are used to stuff cushions for commercial purpose. *M. foetida* is preferred to be grazed by cattle (Kuete et al., 2010; Mada et al., 2013) and is said to be good supplement of protein (Talukdar and Hossain, 2014). The fruits have the essential oils they have been used as insecticides, food additives and aromatherapy (Dandawate et al., 2016).

### *Momordica balsamina* L.

*M. balsamina* is regarded as the most important medicinal plants popularly used as a source of life saving drugs that is most important to the world’s population (Hassan and Umar, 2006; Thakur et al., 2009; Kumar and Bhowmik, 2010; Tan C. et al., 2015; Souda et al., 2018). The crop can be consumed as vegetable in order to supply protein and potassium supplement for diets in poor rural communities, however its high potassium content is known for the executive of hypertension and other cardiovascular conditions (Rahmatullah et al., 2012; Semenya and Potgieter, 2015; Jabeen and Khanum, 2017; Souda et al., 2018). Some of the structures of compounds extracted from *M. balsamina* are listed in Table 3.

**TABLE 3:** Compounds extracted from *Momordica balsamina* L. and their health benefits.

Compound	Structure	Health benefits	References
Cucurbitane glycoside	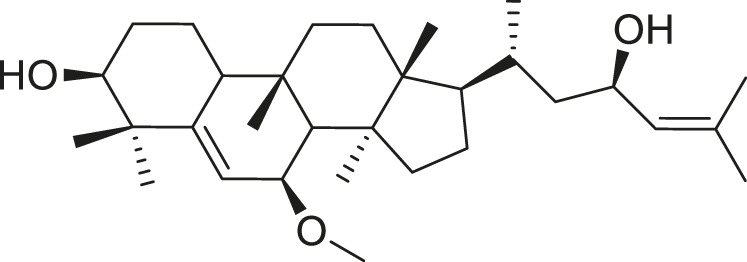	Anti-diabetic	Spengler et al. (2009), Farooq et al. (2019)
Kuguacin J	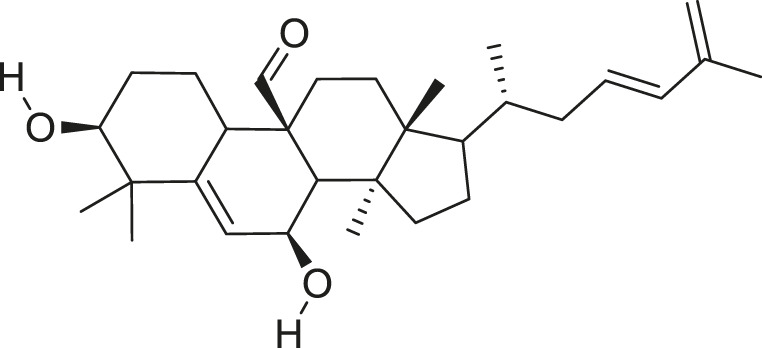	Anti-cancer	Spengler et al. (2009), Santos et al. (2012)
Karavilagenin C	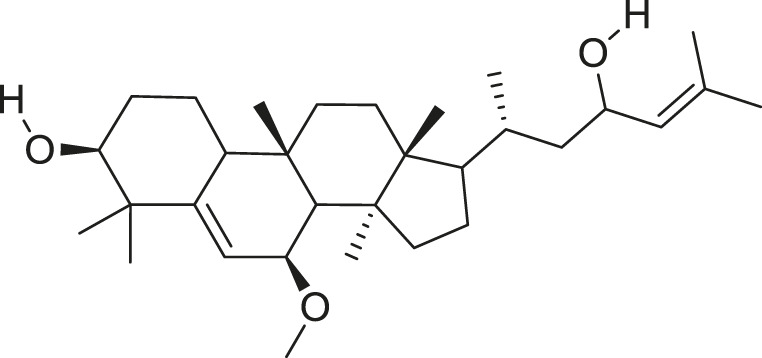	Anti-cancer	Ramalhete et al. (2009), Ma et al. (2012)
Balsaminagenin A	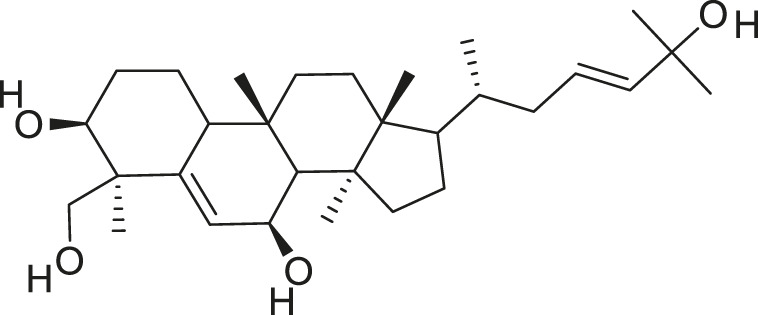	Anti-cancer	Ramalhete et al. (2009), Ma et al. (2012)
Balsaminoside B	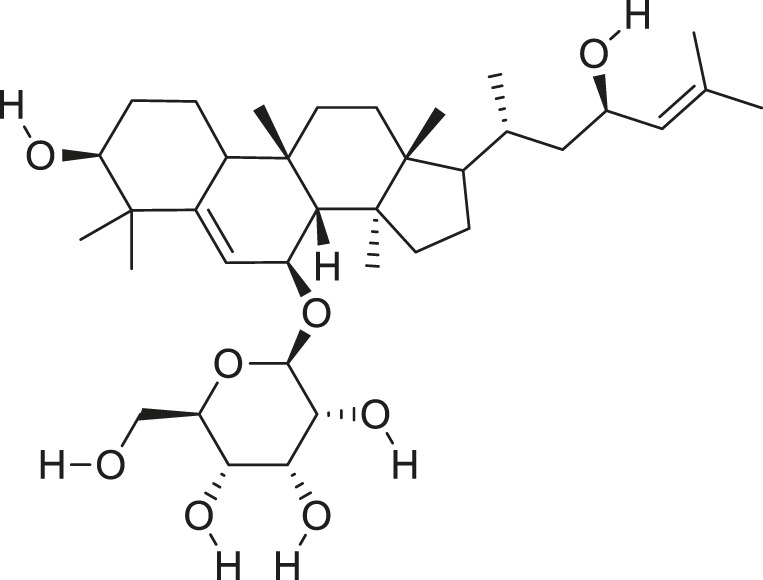	Anti-cancer	Ramalhete et al. (2009), Ma et al. (2012)
Balsaminol F	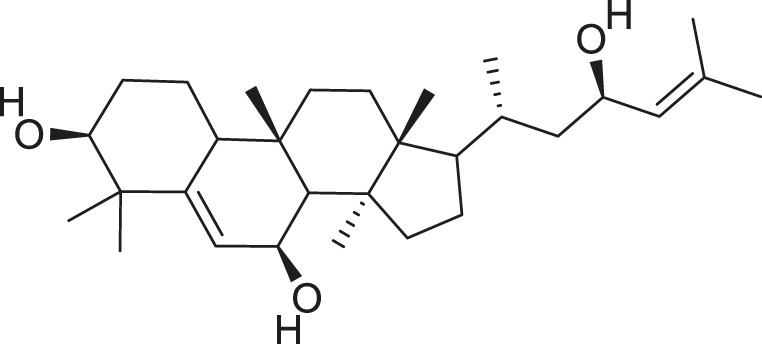	Schistosomicidal activity against *Schistosoma mansoni* adult worms	Ramalhete et al. (2011), Ramalhete et al. (2016)
Momordin I	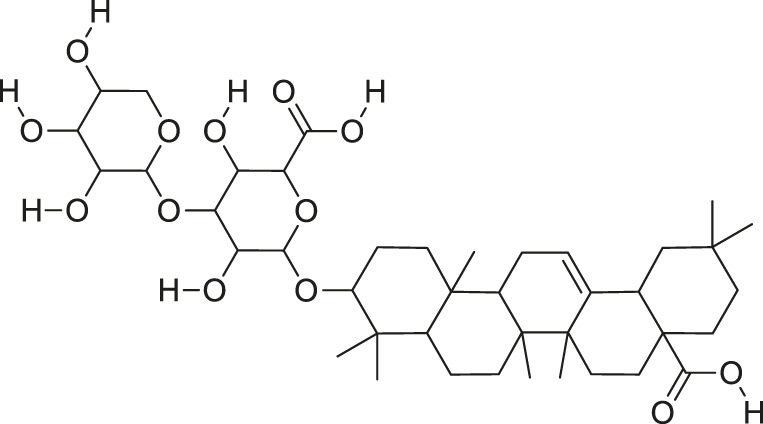	Anti-viral	Thakur et al. (2009)
Momordin II	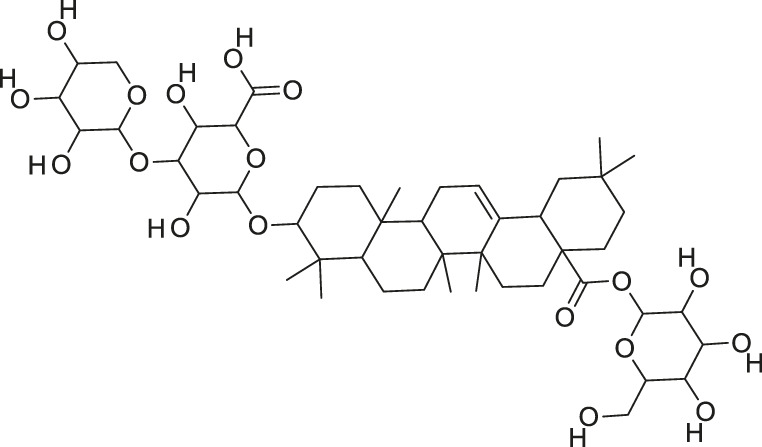	Anti-viral	Thakur et al. (2009)
Balsaminapentaol	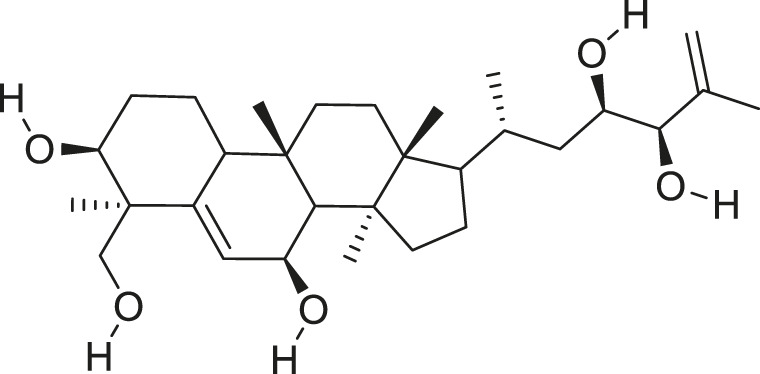	Anti-malarial	Ramalhete et al. (2009), Ma et al. (2012)
Cucurbalsaminol	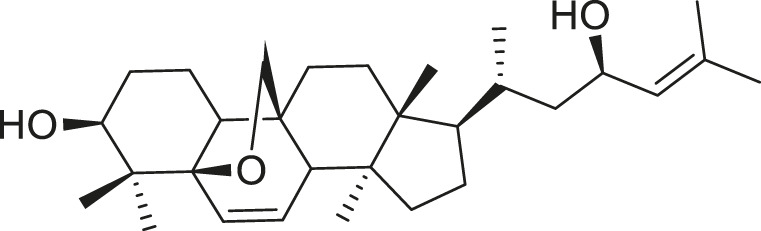	Anti-parasitic	Ma et al. (2012), Shah et al. (2014)

In some parts of Africa and Europe, soaked leaves of *M. balsamina* are used to treat wounds. Furthermore the fruits and leaves are used for treatment of wounds in Nigeria and Syria as hemostatic anti-septic (Mada et al., 2013; Semenya and Potgieter, 2015; Madala et al., 2016). The fruit of *M. balsamina* is used as liniment because of its strong smell and mixed with olive oil and almond oil to treat chapped hands, band and hemorrhoids (Mada et al., 2013). In West Africa *M. balsamina* fruit pulp is mixed with oil as an anti-phlogistic dressing. Mashed fruit of *M. balsamina* is utilized as poultice and bitter tonic (Semenya and Potgieter, 2015). *M. balsamina* plant is used as a medicine for treatment of fevers and yaws. However, Tsonga and Zulus have used leaves as tea for blood-liver deficiencies, stomach and intestinal ailments (Kumar and Bhowmik, 2010).

The Portuguese recommended *M. balsamina* leaves for blood, stomach, and liver deficiencies, furthermore they use leaves for herbal medicine and culinary herb (Thakur et al., 2009; Madala et al., 2014). *M. balsamina* leaves are used for diabetes, digestion disorder, fevers, ulcers, and mild form of malaria. Additionally, in West Africa *M. balsamina* is used as medicine in human and animals particular for fever, yaws and purgative (Oloyede and Aluko, 2012; Ingle et al., 2017; Jabeen and Khanum, 2017; Mostafa et al., 2018). *M. balsamina* roots are used as ingredient in an aphrodisiac preparation and in treatment of urethral discharge on both human and animals. It is advisable to use the whole *M. balsamina* parts of the plant for used in treating skin disease such as scabies (Waako et al., 2005; Ozusaglam and Karakoca, 2013; Pauliuc and Botau, 2013; Wikaningtyas and Sukandar, 2016). In South Africa, mostly the Pedi people use *M. balsamina* leaves and tendrils as potherb and anti-emetic, while the Venda people used the leaves infusions as anti-emetic and Congo as colic. *M. balsamina* leaves and fruit are widely used in Okavango delta for medicinal and spiritual purpose and also as skin aliments in China (Molehin and Adefegha, 2014). In some countries seed can be used as poison in arrow for hunting. The whole plant extract has insecticidal properties (Trakoon-osot et al., 2013; Wardhani et al., 2015; Wang et al., 2016).

The aqueous leaf extract of *M. balsamina* has also been used to minimize and relieve period pain in young girls and postnatal women used it to stimulate milk production (Tan et al., 2008). In Hausa land of Nigeria and Republic of Niger, the leaves are cooked as part of green vegetables soup for post-natal women, where it is believed to help the mother to restore her lost blood during labor and to cleanse her breast milk (Molehin and Adefegha, 2014).

Anti-oxidants are recognized as ingredient and supplement of good human health because they maintain health and prevent diseases such as cancer, coronary heart disease and even altitude sickness in human and animals (Tan et al., 2008; Ingle et al., 2017; Puškárová et al., 2017). *M. balsamina* is used for treatment of diabetes, malaria, colic, sores and wounds, infections, worms and parasites, as an emmenagogue, measles, and hepatitis, fevers, diarrheal, cancer, ulcer, HIV, bacterial infection, and constipation (Semenya and Potgieter, 2015; Abegunde et al., 2018; Aryanti and Lamdayani, 2021). In addition, *M. balsamina* is believed to treat mental illness (Anjamma and Bhavani, 2018). The anti-HIV properties are found at the fruit pulp of *M. balsamina* (Abidemi, 2013). The leaves and fruit extracts of *M. balsamina* contain anti-plasmodial activity against malaria (Balouiri et al., 2016). The crude extract of protein on African pumpkin seeds contains a hemagglutinating activity attributed by the presence of sugar D-galactose and lactose (Bulbul, 2016). Moreover, several previous studies did test the hemagglutinating activity on human and animals of the different blood type and proved that the hemagglutinating was greater toward O blood type compared to other blood cell type (Scorzoni et al., 2007; Souda et al., 2018).

The fruit juice of *M. balsamina* exerted hypoglycemic activity, stimulates appetite and helps in the entire digestion process (Kumar and Bhowmik, 2010; Chen et al., 2011). Hypoglycemic potential of *M. balsamina* has been established in normal and diabetic rats and in patients with type 2 diabetes (Waako et al., 2005; Scorzoni et al., 2007; Leung et al., 2009; Kumar and Bhowmik, 2010; Rahmatullah et al., 2012; Joseph and Jini, 2013; Trakoon-osot et al., 2013; Ludidi et al., 2019). Extract of P-Insulin, polypeptide from the fruits and seeds of *M. balsamina* rapidly reduces and normalized the blood sugar level in rats.

The leaves have been found to be highly hemolytic and hepatotoxic in rats while the fruits are regarded as toxic to various organs and tissues of rats in very high dose (Semenya and Potgieter, 2015). Thus, it is important to determine the correct dose to optimize its potential use as a medicinal plant.

## Conclusion and Future Perspective

**FIGURE 2:**
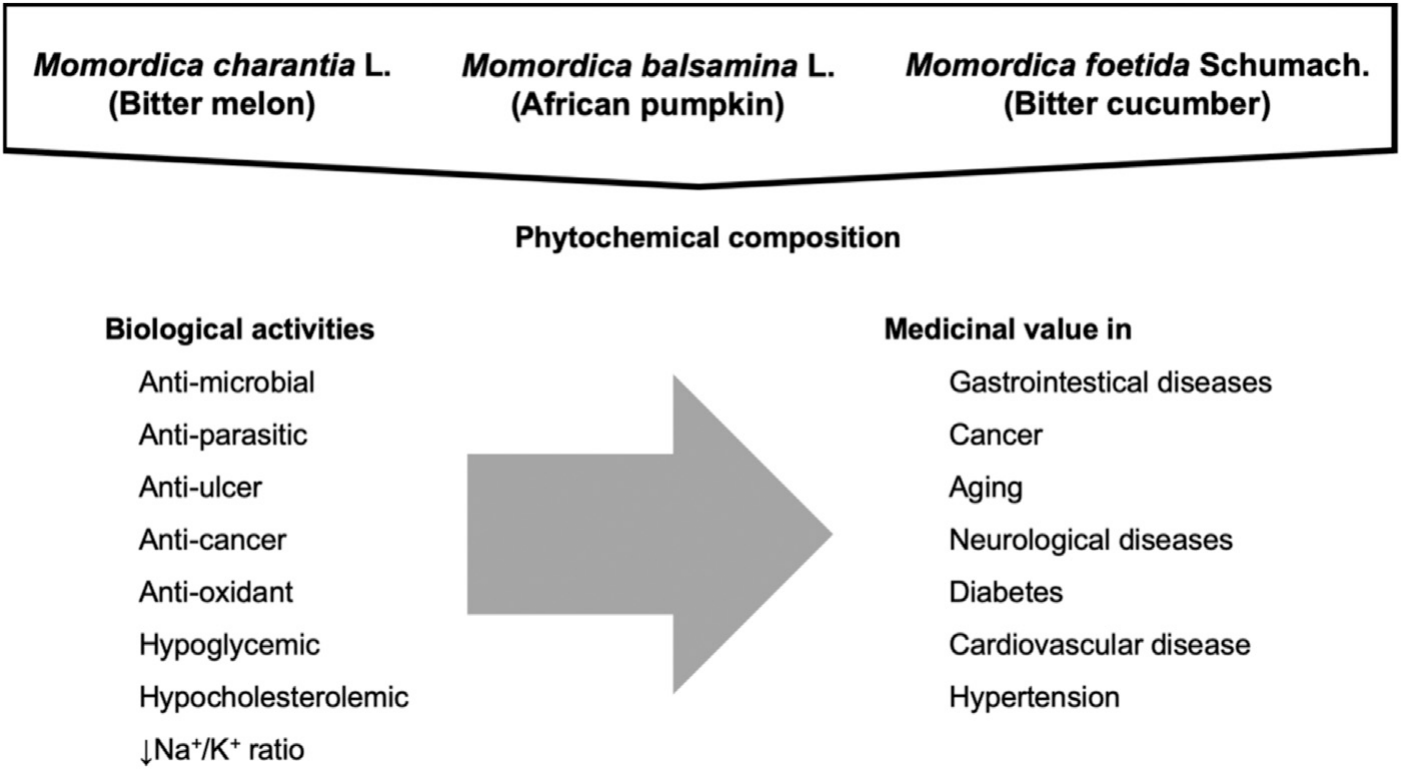
Summarized biological activies and medicinal value of *Momordica charantia* L. *Momordica foetida* Schumach. and *Momordica balsamina* L. (African pumpkin).

The biochemical composition of *M. charantia*, *M. foetida* and *M. balsamina* gives them a great and interesting nutritional and medicinal value. Many of its components have singular biological activity and the synergy of them may exert interesting pharmacological properties. Several studies demonstrated that these important plants have the potential to be utilized for medicinal purposes as they exhibit anti-diabetic, anti-microbial, anthelmintic, abortifacient, anti-bacterial, anti-viral, and chemo-preventive activities. These species possess a promising and innovative source of natural bioactive agents such as resins, alkaloids, flavonoids, glycosides, steroids, terpenes, cardiac glycoside, saponins, pectin, carbohydrates, amino acids, proteins, fats, fiber, chlorophyll, phosphorus, calcium, potassium, magnesium, sodium, zinc, manganese, and iron. The main biological activities of Momordica spp. has been summarized in Figure 2.

The anti-microbial, anti-parasitic and anti-ulcer activities of *Momordica* spp. are the mechanisms related with their inhibitory effects against gastrointestinal diseases. In addition, anti-cancer effects of several biochemicals isolated from *Momordica* spp. has been described. Anti-oxidant activities of *Momordica* spp. also prevent cancer diseases avoiding oxidative stress and oxidative damage such as lipid peroxidation. Moreover, anti-oxidants exerts anti-aging and neuroprotection effects. Additionally, the hypoglycemic activities of *Momordica* spp. exert an anti-diabetes effect, the hypocholesterolemic activities exerts a cardioprotective effect, and their low ratio of Na^+^/K^+^ exerts beneficial effects against hypertension. In addition to being able to be consumed as food or supplement, clinical evidence suggests their effectiveness in managing various ailments such as dysmenorrhea, eczema, emmenagogue, galactagogue, gout, jaundice, kidney (stone), leprosy, leucorrhea, pneumonia, psoriasis, rheumatism, scabies, piles, cancer, coronary heart disease diabetes, digestion disorder, fevers, ulcers, malaria, tumors, asthma, skin infections, and hypertension.

Thus, the review suggests that the three selected crops have medicinal and herbal properties that can be used for various ailments. A better biochemical characterization is needed to better understand of medicinal properties of these plants. This review also assists to understand the commercial properties of the crop since the cultivation as commercial vegetable is currently underway. A better understand of pharmacological activities of *Momortica* spp. will give added value to these plants and benefit the producers.

## References

[B1] Abegunde M. T. Akinpelu D. A. Omololu-Aso J. Otusanya O. O. Akinlolu J. T. (2018). Determination of Antimicrobial, Antioxidant and Phytochemical Properties of *Cocos Nucifera* Linn Endocarp Extract on Bacteria Associated with Human Infection. J. Pharm. Microbiol. 4.

[B2] Abidemi O. O. (2013). Phytochemicals and Spectrophotometric Determination of Metals in Various Medicinal Plants in Nigeria. Int. J. Eng. Sci. Invent 2, 51–54.

[B3] Acquaviva R. Di Giacomo C. Vanella L. Santangelo R. Sorrenti V. Barbagallo I. (2013). Antioxidant Activity of Extracts of Momordica Foetida Schumach. et Thonn. Molecules 18, 3241–3249. 10.3390/molecules18033241 23486103 PMC6269981

[B4] Aeri V. Raj R. (2020). The Bitter Gourd Genome. Cham: Springer, 33–44. 10.1007/978-3-030-15062-4_3 . Medicinal Properties of Bitter Gourd: Bioactives and Their Actions

[B5] Anilakumar K. R. Kumar G. P. Ilaiyaraja N. (2015). Nutritional, Pharmacological and Medicinal Properties of M*omordica Charantia* . Int. J. Food Sci. Nutr. 4, 75–83.

[B6] Anjamma M. Bhavani N. L. (2018). Comparative Antibacterial and Antioxidant Activity from Root and Fruit Extracts of *Momordica Charantia* L . And Momordica Dioica Roxb. Int. J. S Res. Sci. Tech. 4, 1710–1716.

[B7] Aparicio H. Hedberg I. Bandeira S. Ghorbani A. (2021). Ethnobotanical Study of Medicinal and Edible Plants Used in Nhamacoa Area, Manica Province-Mozambique. South Afr. J. Bot. 139, 318–328. 10.1016/j.sajb.2021.02.029

[B8] Aryanti A. Lamdayani R. (2021). The Effect of Fraction and Active Compounds of Momordica Balsamina L. On Bacteria *Salmonella* Typhi Causing Salmonellosis. Ijghr 3 (1), 29–42. 10.37287/ijghr.v3i1.291

[B9] Asna A. C. Joseph J. Joseph John K. (2020). The Bitter Gourd Genome. Cham: Springer, 7–31. 10.1007/978-3-030-15062-4_2 . Botanical Description of Bitter Gourd

[B10] Badhwar R. Kaur G. Popli H. Yadav D. Buttar H. S. (2020). “Pathophysiology of Obesity-Related Non-communicable Chronic Diseases and Advancements in Preventive Strategies,” in Pathophysiology of Obesity-Induced Health Complications. Editors Tappia P. S. Ramjiawan B. Dhalla N. S. (Cham: Springer International Publishing)), 317–340. 10.1007/978-3-030-35358-2_19

[B11] Bakare R. I. Magbagbeola O. A. Akinwande A. I. Okunowo O. W. (2010). Nutritional and Chemical Evaluation of *Momordica Charantia* . J. Med. Plant Res. 4, 2189–2193.

[B12] Balouiri M. Sadiki M. Ibnsouda S. K. (2016). Methods for In Vitro Evaluating Antimicrobial Activity: A Review. J. Pharm. Anal. 6, 71–79. 10.1016/j.jpha.2015.11.005 29403965 PMC5762448

[B13] Beloin N. Gbeassor M. Akpagana K. Hudson J. De Soussa K. Koumaglo K. (2005). Ethnomedicinal Uses of *Momordica Charantia* (Cucurbitaceae) in Togo and Relation to its Phytochemistry and Biological Activity. J. Ethnopharmacology 96, 49–55. 10.1016/j.jep.2004.08.009 15588650

[B14] Birla D. (2016). Evaluation of Antibacterial Activity of *Momordica Charantia* . Pharma Tutor 4, 37–40.

[B15] Bishayee A. Ahmed S. Brankov N. Perloff M. (2011). Triterpenoids as Potential Agents for the Chemoprevention and Therapy of Breast Cancer. Front. Biosci. 16, 980–996. 10.2741/3730 PMC3057757 21196213

[B16] Braca A. Siciliano T. D’Arrigo M. Germanò M. P. (2008). Chemical Composition and Antimicrobial Activity of *Momordica Charantia* Seed Essential Oil. Fitoterapia 79, 123–125. 10.1016/j.fitote.2007.11.002 18164872

[B17] Bulbul I. J. (2016). Determination of Antibacterial, Antifungal and Cytotoxic Activities of N-Hexane, Chloroform and Ethyl Acetate Extracts of *Momordica Charantia* Leaves. PharmaTutor 4, 28–33.

[B18] Cantwell M. Nie X. Zong R. J. Yamaguchi M. (1996). Progress in New Crops. Arlington: ASHS Press, 488–495.Asian Vegetables: Selected Fruit and Leafy Types

[B19] Cao X. Sun Y. Lin Y. Pan Y. Farooq U. Xiang L. (2018). Antiaging of Cucurbitane Glycosides from Fruits of *Momordica Charantia* L. Oxid Med. Cel Longev 2018, 1538632. 10.1155/2018/1538632 PMC5889887 29765490

[B20] Chen F. Huang G. Yang Z. Hou Y. (2019). Antioxidant Activity of *Momordica Charantia* Polysaccharide and its Derivatives. Int. J. Biol. Macromolecules 138, 673–680. 10.1016/j.ijbiomac.2019.07.129 31344411

[B21] Chen J.-C. Lau C. Chan J. Fung K.-P. Leung P.-C. Liu J.-Q. (2015). The Antigluconeogenic Activity of Cucurbitacins from Momordica Charantia. Planta Med. 81, 327–332. 10.1055/s-0035-1545695 25760384

[B22] Chen Q. Chan L. L. Y. Li E. T. S. (2003). Bitter Melon (*Momordica Charantia*) Reduces Adiposity, Lowers Serum Insulin and Normalizes Glucose Tolerance in Rats Fed a High Fat Diet. J. Nutr. 133, 1088–1093. 10.1093/jn/133.4.1088 12672924

[B23] Chen Y. F. Roan H. Y. Lii C. K. Huang Y. C. Wang T. S. (2011). Relationship between Antioxidant and Antiglycation Ability of Saponins, Polyphenols, and Polysaccharides in Chinese Herbal Medicines Used to Treat Diabetes. J. Med. Plant Res. 5, 2322–2331.

[B24] Cohen M. Huband M. D. Yoder S. L. Gage J. W. Roland G. E. (1998). Bacterial Eradication by Clinafloxacin, CI-990, and Ciprofloxacin Employing MBC Test, In-Vitro Time-Kill and In-Vivo Time-Kill Studies. J. Antimicrob. Chemother. 41, 605–614. 10.1093/jac/41.6.605 9687098

[B25] Costa J. G. Nascimento E. M. Campos A. R. Rodrigues F. F. (2010). Antibacterial Activity of *Momordica Charantia* (Curcubitaceae) Extracts and Fractions. J. Basic Clin. Pharm. 2, 45–51. 24826002 PMC3979203

[B26] Dabeek W. M. Marra M. V. (2019). Dietary Quercetin and Kaempferol: Bioavailability and Potential Cardiovascular-Related Bioactivity in Humans. Nutrients 11, 2288. 10.3390/nu11102288 PMC6835347 31557798

[B27] Dandawate P. R. Subramaniam D. Padhye S. B. Anant S. (2016). Bitter Melon: a Panacea for Inflammation and Cancer. Chin. J. Nat. medicines 14, 81–100. 10.1016/s1875-5364(16)60002-x PMC5276711 26968675

[B28] Daniel P. Supe U. Roymon M. G. (2014). A Review on Phytochemical Analysis of *Momordica Charantia* . Int. J. Adv. Pharm. Biol. Chem. 3, 214–220.

[B29] Dellavalle P. D. Cabrera A. Alem D. Larrañaga P. Ferreira F. Dalla Rizza M. (2011). Antifungal Activity of Medicinal Plant Extracts against Phytopathogenic Fungus *Alternaria* Spp. Chilean J. Agric. Res. 71, 231–239. 10.4067/S0718-58392011000200008

[B30] Desai S. Tatke P. (2015). Charantin: An Important Lead Compound from *Momordica Charantia* for the Treatment of Diabetes. J. Pharmacogn Phytochem. 3, 163–166.

[B31] Devyani B. Chakravarthy A. Mutalik S. Devkar R. (2016). Determination of Antibacterial and Antifungal Properties of Rose Extract- an In Vitro Study. Int. J. Pharmacogn Phytochem. Res. 8, 1695–1697.

[B32] Dzotam J. K. Touani F. K. Kuete V. (2016). Antibacterial Activities of the Methanol Extracts of *Canarium Schweinfurthii* and Four Other Cameroonian Dietary Plants against Multi-Drug Resistant Gram-Negative Bacteria. Saudi J. Biol. Sci. 23, 565–570. 10.1016/j.sjbs.2015.06.006 27579004 PMC4992100

[B33] Farooq U. Pan Y. Lin Y. Wang Y. Osada H. Xiang L. (2019). Structure Characterization and Action Mechanism of an Antiaging New Compound from *Gastrodia Elata* Blume. Oxidative Med. Cell Longevity 2019, 5459862. 10.1155/2019/5459862 PMC6526511 31198492

[B34] Fongod A. G. N. Ngoh L. M. Veranso M. C. (2014). Ethnobotany, Indigenous Knowledge and Unconscious Preservation of the Environment: An Evaluation of Indigenous Knowledge in South and Southwest Regions of Cameroon. Int. J. Biodivers. Conserv. 6, 85–99. 10.5897/ijbc2013.0637

[B35] Froelich S. Onegi B. Kakooko A. Siems K. Schubert C. Jenett-Siems K. (2007). Plants Traditionally Used against Malaria: Phytochemical and Pharmacological Investigation of *Momordica Foetida* . Rev. Bras. Farmacogn. 17, 1–17. 10.1590/s0102-695x2007000100002

[B36] Ghosh D. (2014). Does Bitter Melon (*Momordica Charantia*) Have Antibacterial Property? J. Food Process. Technol. 5, 1000345. 10.4172/2157-7110.1000345

[B37] Grover J. K. Yadav S. P. (2004). Pharmacological Actions and Potential Uses of Momordica Charantia: a Review. J. Ethnopharmacology 93, 123–132. 10.1016/j.jep.2004.03.035 15182917

[B38] Gupta M. Sharma S. Gautam A. Bhadauria R. (2011). *Momordica Charantia* Linn. (Karela): Nature's Silent Healer. Int. J. Pharm. Sci. Rev. Res. 11, 32–37.

[B39] Gupta P. (2020). “Targeted Cancer Therapy with Bioactive Foods and Their Products,” in Functional Foods in *Cancer* Prevention and Therapy. Editor Kabir Y. (Academic Press), 33–46. 10.1016/b978-0-12-816151-7.00002-8

[B40] Harinantenaina L. Tanaka M. Takaoka S. Oda M. Mogami O. Uchida M. (2006). *Momordica Charantia* Constituents and Antidiabetic Screening of the Isolated Major Compounds. Chem. Pharm. Bull. 54, 1017–1021. 10.1248/cpb.54.1017 16819222

[B41] Hassan L. G. Umar K. J. (2006). Nutritional Value of Balsam Apple (M*omordica Balsamina* L.) Leaves. Pak J. Nutr. 5, 525–529. 10.3923/pjn.2006.522.529

[B42] Huang H.-L. Hong Y.-W. Wong Y.-H. Chen Y.-N. Chyuan J.-H. Huang C.-J. (2008). Bitter Melon (*Momordica Charantia* L.) Inhibits Adipocyte Hypertrophy and Down Regulates Lipogenic Gene Expression in Adipose Tissue of Diet-Induced Obese Rats. Br. J. Nutr. 99, 230–239. 10.1017/s0007114507793947 17651527

[B43] Huang H. Chen F. Long R. Huang G. (2020). The Antioxidant Activities In Vivo of Bitter Gourd Polysaccharide. Int. J. Biol. Macromolecules 145, 141–144. 10.1016/j.ijbiomac.2019.12.165 31870875

[B44] Ibrahim M. A. Saeed B. O. Elsaid Konozy E. H. Ahmed S. M. Mohamed M. E. (2014). Isolation and Purification of Lection from *Momordica Balsamina* Seeds. Am. Int. J. Contem Res. 2, 4.

[B45] Ingle K. P. Deshmukh A. G. Padole D. A. Dudhare M. S. Moharil M. P. Khelurkar V. C. (2017). Phytochemicals: Extraction Methods, Identification and Detection of Bioactive Compounds from Plant Extracts. J. Pharmacogn Phytochem. 6, 32–36.

[B46] Islam S. Jalaluddin M. (2019). Biological Functions and Sensory Attributes of Different Skin Colored Bitter Melon (*Momordica Charantia* L.) Varieties. Am. J. Food Sci. H 5, 25–31.

[B47] Jabeen U. Khanum A. (2017). Isolation and Characterization of Potential Food Preservative Peptide from *Momordica Charantia* L. Arabian J. Chem. 10, S3982–S3989. 10.1016/j.arabjc.2014.06.009

[B48] Jia S. Shen M. Zhang F. Xie J. (2017). Recent Advances in *Momordica Charantia*: Functional Components and Biological Activities. Ijms 18, 2555. 10.3390/ijms18122555 PMC5751158 29182587

[B49] Jiang Y. Peng X.-R. Yu M.-Y. Wan L.-S. Zhu G.-L. Zhao G.-T. (2016). Cucurbitane-type Triterpenoids from the Aerial Parts of *Momordica Charantia* L. Phytochemistry Lett. 16, 164–168. 10.1016/j.phytol.2016.04.007

[B50] Joseph B. Jini D. (2013). Antidiabetic Effects of *Momordica Charantia* (Bitter Melon) and its Medicinal Potency. Asian Pac. J. Trop. Dis. 3, 93–102. 10.1016/s2222-1808(13)60052-3

[B51] Ju Y. H. Clausen L. M. Allred K. F. Almada A. L. Helferich W. G. (2004). β-Sitosterol, β-Sitosterol Glucoside, and a Mixture of β-Sitosterol and β-Sitosterol Glucoside Modulate the Growth of Estrogen-Responsive Breast *Cancer* Cells *In Vitro* and in Ovariectomized Athymic Mice. J. Nutr. 134, 1145–1151. 10.1093/jn/134.5.1145 15113961

[B52] Kandakumar S. Manju D. V. (2017). Pharmacological Applications of Isorhamnetin: A Short Review. Ijtsrd Volume-1, 672–678. 10.31142/ijtsrd2202

[B53] Kangsamaksin T. Chaithongyot S. Wootthichairangsan C. Hanchaina R. Tangshewinsirikul C. Svasti J. (2017). Lupeol and Stigmasterol Suppress Tumor Angiogenesis and Inhibit Cholangiocarcinoma Growth in Mice via Downregulation of Tumor Necrosis Factor-Alpha. PLoS One 12, e0189628. 10.1371/journal.pone.0189628 29232409 PMC5726636

[B54] Kaur N. Chaudhary J. Jain A. Kishore L. (2011). Stigmasterol: a Comprehensive Review. J. Pharm. Sci. Res. 2, 2259.

[B55] Kesari P. Pratap S. Dhankhar P. Dalal V. Mishra M. Singh P. K. (2020). Structural Characterization and In-Silico Analysis of *Momordica Charantia* 7S Globulin for Stability and ACE Inhibition. Scientific Rep. 10, 1160. 10.1038/s41598-020-58138-9 PMC6981215 31980708

[B56] Khadijeh S. Elham J. Mahboubeh M. Mehdi H. (2019). Study the Antimicrobial Effects of *Momordica Charantia* on Pathogenic Bacteria. J. Med. Bacteriol. 8, 1–7.

[B57] Khalid Z. Hassan S. M. Mughal S. S. Hassan S. K. Hassan H. (2021). Phenolic Profile and Biological Properties of *Momordica Charantia* . Chem. Biomol. Eng. 6 (1), 17–29. 10.11648/j.cbe.20210601.13

[B58] Khanna P. (2004). Protein/polypeptide-k Obtained from *Momordica Charantia* and a Process for the Extraction Thereof. U.S. Patent 6, 831. *162*.

[B59] Khoza B. S. Dubery I. A. Byth-Illing H.-A. Steenkamp P. A. Chimuka L. Madala N. E. (2016). Optimization of Pressurized Hot Water Extraction of Flavonoids from *Momordica Foetida* Using UHPLC-qTOF-MS and Multivariate Chemometric Approaches. Food Anal. Methods 9, 1480–1489. 10.1007/s12161-015-0302-8

[B60] Kim K. B. Lee S. Kang I. Kim J. H. (2018). *Momordica Charantia* Ethanol Extract Attenuates H(2)O(2)-Induced Cell Death by its Antioxidant and Anti-apoptotic Properties in Human Neuroblastoma SK-N-MC Cells. Nutrients 10. 10.3390/nu10101368 PMC6213776 30249986

[B61] Koneri R. Balaraman R. Saraswati C. D. (2006). Antiovulatory and Abortifacient Potential of the Ethanolic Extract of Roots of *Momordica Cymbalaria* Fenzl in Rats. Indian J. Pharmacol. 38. 10.4103/0253-7613.24616

[B62] Krawinkel M. B. Keding G. B. (2006). Bitter Gourd (Momordica Charantia):A Dietary Approach to Hyperglycemia. Nutr. Rev. 64, 331–337. 10.1111/j.1753-4887.2006.tb00217.x 16910221

[B63] Kuete V. Poumale H. M. P. Guedem A. N. Shiono Y. Randrianasolo R. Ngadjui B. T. (2010). Antimycobacterial, Antibacterial and Antifungal Activities of the Methanol Extract and Compounds from *Thecacoris Annobonae* (Euphorbiaceae). South Afr. J. Bot. 76, 536–542. 10.1016/j.sajb.2010.04.003

[B64] Kulkarni P. Lohidasan S. Mahadik K. (2021). Isolation, Characterisation and Investigation of In Vitro Antidiabetic and Antioxidant Activity of Phytoconstituents from Fruit of *Momordica Charantia* Linn. Nat. Product. Res. 35 (6), 1035–1037. 10.1080/14786419.2019.1613400 31264459

[B65] Kumar K. S. Bhowmik D. (2010). Traditional Medicinal Uses and Therapeutic Benefits of *Momordica Charanti*a Linn. Int. J. Pharm. Sci. Rev. Res. 4, 23–28. 10.4103/0973-7847.65327

[B66] Kumar R. Balaji S. Uma T. S. Sehgal P. K. (2009). Fruit Extracts of Momordica Charantia Potentiate Glucose Uptake and Up-Regulate Glut-4, PPARγ and PI3K. J. Ethnopharmacology 126, 533–537. 10.1016/j.jep.2009.08.048 19744549

[B67] Lee S. H. Jeong Y. S. Song J. Hwang K.-A. Noh G. M. Hwang I. G. (2017). Phenolic Acid, Carotenoid Composition, and Antioxidant Activity of Bitter Melon (*Momordica Charantia* L.) at Different Maturation Stages. Int. J. Food Properties 20, S3078–S3087. 10.1080/10942912.2016.1237961

[B68] Lee Y. H. Yoon S.-Y. Baek J. Kim S. J. Yu J. S. Kang H. (2021). Metabolite Profile of Cucurbitane-type Triterpenoids of Bitter Melon (Fruit of *Momordica Charantia*) and Their Inhibitory Activity against Protein Tyrosine Phosphatases Relevant to Insulin Resistance. J. Agric. Food Chem. 69 (6), 1816–1830. 10.1021/acs.jafc.0c06085 33406828

[B69] Leelaprakash G. Rose J. C. Gowtham B. M. Javvaji P. K. Prasad S. A. (2011). *In vitro* antimicrobial and Antioxidant Activity of *Momordica Charantia* Leaves. Pharmacophore 2, 244–252.

[B70] Leung L. Birtwhistle R. Kotecha J. Hannah S. Cuthbertson S. (2009). Anti-diabetic and Hypoglycaemic Effects of *Momordica Charantia* (Bitter Melon): a Mini Review. Br. J. Nutr. 102, 1703–1708. 10.1017/s0007114509992054 19825210

[B71] Li Q. Y. Liang H. Wang B. Zhao Y. Y. (2009). [Chemical Constituents of *Momordica Charantia* L]. Yao Xue Xue Bao 44, 1014–1018. 20055177

[B72] Li W. Lin Z. Yang C. Wang Y. Qiao Y. (2015).Study on the Chemical Constituents of *Momordica Charantia* L. Leaves Andmethod for Their Quantitative Determination. Biomed. Research-tokyo, 26, 415–419.

[B73] Lii C.-K. Chen H.-W. Yun W.-T. Liu K.-L. (2009). Suppressive Effects of Wild Bitter Gourd (Momordica Charantia Linn. Var. Abbreviata ser.) Fruit Extracts on Inflammatory Responses in RAW 264.7 Macrophages. J. Ethnopharmacology 122, 227–233. 10.1016/j.jep.2009.01.028 19330915

[B74] Limtrakul P. Pitchakarn P. Suzuki S. (2013). Kuguacin J, a Triterpenoid from *Momordica Charantia* Linn: A Comprehensive Review of Anticarcinogenic Properties. Carcinogenesis 275. 10.5772/55532

[B75] Liu J.-Q. Chen J.-C. Wang C.-F. Qiu M.-H. Liu Z. Gong J. (20092021). New Cucurbitane Triterpenoids and Steroidal Glycoside from *Momordica charanti*aThe Effect of Momordica Charantia in the Treatment of Diabetes Mellitus: A Review. Moleculesevid Based Complement. Altern Med 142021, 4804–4813. 10.3390/molecules14124804

[B76] Lu Y.-L. Liu Y. H. Liang W. L. Chuang J. H. Cheng K.-T. Liang H. J. (2011). Antibacterial and Cytotoxic Activities of Different Wild Bitter Gourd Cultivars (*Momordica Charantia* L. Var. Abbreviata Seringe). Bot. Stud. 52, 427–434.

[B77] Ludidi A. Baloyi M. C. Khathi A. Sibiya N. H. Ngubane P. S. (2019). The Effects of *Momordica Balsamina* Methanolic Extract on Haematological Function in Streptozotocin-Induced Diabetic Rats: Effects on Selected Markers. Biomed. Pharmacother. 116, 108925. 10.1016/j.biopha.2019.108925 31112874

[B78] Ma J. Krynitsky A. J. Grundel E. Rader J. I. (2012). Quantitative Determination of Cucurbitane-type Triterpenes and Triterpene Glycosides in Dietary Supplements Containing Bitter Melon (*Momordica Charantia*) by HPLC-MS/MS. J. AOAC Int. 95, 1597–1608. 10.5740/jaoacint.11-511 23451374

[B79] Mada S. B. Garba A. Mohammed H. a. A. Muhammad A. Olagunju A. (2013). Antimicrobial Activity and Phytochemical Screening of Aqueous and Ethanol Extracts of *Momordica Charantia* L. Leaves. J. Med. Plants Res. 7, 579–586.

[B80] Madala N. E. Tugizimana F. Steenkamp P. A. (2014). Development and Optimization of an UPLC-QTOF-MS/MS Method Based on an In-Source Collision Induced Dissociation Approach for Comprehensive Discrimination of Chlorogenic Acids Isomers from Momordica Plant Species. J. Anal. Methods Chem. 2014, 650879. 10.1155/2014/6508792014 25295221 PMC4177087

[B81] Madala N. E. Piater L. Dubery I. Steenkamp P. (2016). Distribution Patterns of Flavonoids from Three *Momordica* Species by Ultra-high Performance Liquid Chromatography Quadrupole Time of Flight Mass Spectrometry: a Metabolomic Profiling Approach. Revista Brasileira de Farmacognosia 26, 507–513. 10.1016/j.bjp.2016.03.009

[B82] Malik J. A. Iqbal S. Biswas J. Riaz U. Datta S. (2021). Antidiabetic Property of Aloe Vera (*Aloe Barbadensi*s) and Bitter Melon (*Momordica Charantia*). Med. Aromatic Plants: Healthc. Ind. Appl., 257–269. 10.1007/978-3-030-58975-2_10

[B83] Marquis V. Adanlawo T. Olaniyi A. (1977). The Effect of Foetidin from *Momordica Foetida* on Blood Glucose Level of Albino Rats. Planta Med. 31, 367–374. 10.1055/s-0028-1097545 882600

[B84] Moher D. Liberati A. Tetzlaff J. Altman D. G. (2009). Preferred Reporting Items for Systematic Reviews and Meta-Analyses: the PRISMA Statement. Plos Med. 6, e1000097. 10.1371/journal.pmed.1000097 19621072 PMC2707599

[B85] Molehin O. R. Adefegha S. A. (2014). Comparative Study of the Aqueous and Ethanolic Extract of *Momordica Foetida* on the Phenolic Content and Antioxidant Properties. Int. Food Res. J. 21, 401.

[B86] Mostafa A. A. Al-Askar A. A. Almaary K. S. Dawoud T. M. Sholkamy E. N. Bakri M. M. (2018). Antimicrobial Activity of Some Plant Extracts against Bacterial Strains Causing Food Poisoning Diseases. Saudi J. Biol. Sci. 25, 361–366. 10.1016/j.sjbs.2017.02.004 29472791 PMC5815983

[B87] Mulholland D. A. Sewram V. Osborne R. Pegel K. H. Connolly J. D. (1997). Cucurbitane Triterpenoids from the Leaves of *Momordica Foetida* . Phytochemistry 45, 391–395. 10.1016/s0031-9422(96)00814-x

[B88] Mwambete K. D. (2009). The In Vitro Antimicrobial Activity of Fruit and Leaf Crude Extracts of *Momordica Charantia*: a Tanzania Medicinal Plant. Afr. Health Sci. 9, 34–39. 20842240 PMC2932517

[B89] Ndam L. Mih A. Fongod A. Tening A. S. Tonjock R. K. Enang J. (2014). Phytochemical Screening of the Bioactive Compounds in Twenty (20) Cameroonian Medicinal Plants. Int. J. Curr. Microbiol. App Sci. 3, 768–778.

[B90] Nerurkar P. V. Lee Y. K. Nerurkar V. R. (2010). *Momordica Charantia* (Bitter Melon) Inhibits Primary Human Adipocyte Differentiation by Modulating Adipogenic Genes. BMC Complement. Altern. Med. 10, 34. 10.1186/1472-6882-10-34 20587058 PMC2911406

[B91] Nitu S. Patidar K. C. (2017). Evaluation of Antimicrobial Activity Determination and Phytochemical Investigation in Selected Plants. Int. J. Pharmacogn Phytochem. Res. 9, 1429–1434. 10.25258/phyto.v9i11.11187

[B92] Nyam K. L. Tan C. P. Lai O. M. Long K. Che Man Y. B. (2009). Physicochemical Properties and Bioactive Compounds of Selected Seed Oils. LWT - Food Sci. Tech. 42, 1396–1403. 10.1016/j.lwt.2009.03.006

[B93] Odeleye O. M. Oyedeji O. A. Shode F. O. (2009). Constituents of *Momordica Foetida* and Evaluation of Their Antimicrobial Activity. Planta Med. 75, 24. 10.1055/s-2009-1216462 19016407

[B94] Odusote M. O. Awaraka C. N. (2004). *Momordica Foetida* (Cucurbitacea) A Potential Laxative Granule/Capsule. Nig Q. J. Hosp. Med. 14, 189–194. 10.4314/nqjhm.v14i2.12719

[B95] Okabe H. Miyahara Y. Yamauchi T. (1982). Studies on the Constituents of Momordica Charantia L. IV. Characterization of the New Cucurbitacin Glycosides of the Immature Fruits. (2). Structures of the Bitter Glycosides, Momordicosides K and L. Chem. Pharm. Bull. 30, 4334–4340. 10.1248/cpb.30.4334

[B96] Olaniyi A. A. Marquis V. O. (1975). Phytochemical and Preliminary Pharmacological Investigation of the Alkaloid Obtained from *Momordica Foetida* . J. Pharm. 6, 117–119.

[B97] Oloyede O. I. Aluko O. M. (2012). Determination of Antioxidant Potential of *Momordica Foetida* Leaf Extract on Tissue Homogenate. Sci. J. Clin. Med. Trial 2012, 1–4. 10.7237/sjmct/225

[B98] Orhan D. D. Özçelik B. Özgen S. Ergun F. (2010). Antibacterial, Antifungal, and Antiviral Activities of Some Flavonoids. Microbiol. Res. 165, 496–504. 10.1016/j.micres.2009.09.002 19840899

[B99] Oronje M. L. Hagen M. Gikungu M. Kasina M. Kraemer M. (2012). Pollinator Diversity, Behaviour and Limitation on Yield of Karela (*Momordica Charantia* L. Cucurbitaceae) in Western Kenya. Afr. J. Agric. Res. 7, 1629–1638. 10.5897/AJAR11.725

[B100] Ozusaglam M. A. Karakoca K. (2013). Antimicrobial and Antioxidant Activities of *Momordica Charantia* from Turkey. Afr. J. Biotechnol. 12 (13), 1548–1558. 10.5897/AJB2012.2932

[B101] Palada M. C. Chang L. C. (2003). Suggested Cultural Practices for Bitter Gourd. AVRDC Int. Cooperators’ Guide, 3–15.

[B102] Pari M. Arjunan N. Subramani U. Ramasamy V. Kadarkarai M. Chinnathambi V. (2020). Water Purification and Larvicidal Activity of Seed Extract, *Momordica Charantia* . GSC Adv. Res. Rev. 2, 1–9. 10.30574/gscarr.2020.2.1.0001

[B103] Patel S. Patel T. Parmar K. Bhatt Y. Patel Y. Patel N. M. (2010). Isolation, Characterization and Antimicrobial Activity of Charantin from *Momordica Charantia* Linn. Fruit. Int. J. Drug Dev. Res. 2, 629–634. 10.4103/0974-8490.65509

[B104] Patil S. S. Rathod V. K. (2020). Synergistic Effect of Ultrasound and Three Phase Partitioning for the Extraction of Curcuminoids from C*urcuma Longa* and its Bioactivity Profile. Process Biochem. 93, 85–93. 10.1016/j.procbio.2020.02.031

[B105] Pauliuc I. Botau D. (2013). Antibacterial Activity of *Momordica Charantia* L. Gemmotherapic Extract. Scientific Bull. Ser. F Biotechnologies 17, 57–60.

[B106] Perera W. H. Shivanagoudra S. R. Pérez J. L. Kim D. M. Sun Y. K. (2021). Anti-Inflammatory, Antidiabetic Properties and In Silico Modeling of Cucurbitane-type Triterpene Glycosides from Fruits of an Indian Cultivar of Momordica Charantia L. Molecules 26, 1038. 10.3390/molecules26041038 33669312 PMC7920048

[B107] Perumal V. Khatib A. Uddin Ahmed Q. Fathamah Uzir B. Abas F. Murugesu S. (2021). Antioxidants Profile of Momordica Charantia Fruit Extract Analyzed Using LC-MS-QTOF-based Metabolomics. Food Chem. Mol. Sci. 2, 100012. 10.1016/j.fochms.2021.100012 PMC8991829 35415640

[B108] Pinar M. Rico M. Pascual C. Fernández B. (1983). Foetidin, an 8,9-Seco-17-Nor-Kaurane Diterpenoid from Elaeoselinum Foetidum. Phytochemistry 22, 2775–2777. 10.1016/s0031-9422(00)97694-5

[B109] Pirillo A. Verotta L. Gariboldi P. Torregiani E. Bombardelli E. (1995). Constituents of Nothapodytes Foetida. J. Chem. Soc. Perkin Trans. 1 1, 583–587. 10.1039/p19950000583

[B110] Poyraz L. E. Derdovski C. (2016). Morpho-anatomical Investigations on *Momordica Charantia* L. (Cucurbitaceae). Anadolu Univ. J. Sci. Tech. - C. Life Sci. Biotechnol. 5, 23–30. 10.18036/btdc.78679

[B111] Puškárová A. Bučková M. Kraková L. Pangallo D. Kozics K. (2017). The Antibacterial and Antifungal Activity of Six Essential Oils and Their Cyto/genotoxicity to Human HEL 12469 Cells. Sci. Rep. 7, 8211. 10.1038/s41598-017-08673-9 28811611 PMC5557807

[B112] Rahmatullah M. Azam M. N. Khatun Z. Seraj S. Islam F. Rahman M. A. (2012). Medicinal Plants Used for Treatment of Diabetes by the Marakh Sect of the Garo Tribe Living in Mymensingh District, Bangladesh. Afr. J. Tradit Complement. Altern. Med. 9, 380–385. 10.4314/ajtcam.v9i3.12 23983370 PMC3746667

[B113] Raina K. Kumar D. Agarwal R. (2016). Promise of Bitter Melon (Momordica Charantia) Bioactives in Cancer Prevention and Therapy. Semin. Cancer Biol. 40-41, 116–129. 10.1016/j.semcancer.2016.07.002 27452666 PMC5067200

[B114] Ramalhete C. Molnár J. Mulhovo S. Rosário V. E. Ferreira M.-J. U. (2009). New Potent P-Glycoprotein Modulators with the Cucurbitane Scaffold and Their Synergistic Interaction with Doxorubicin on Resistant Cancer Cells. Bioorg. Med. Chem. 17, 6942–6951. 10.1016/j.bmc.2009.08.020 19733087

[B115] Ramalhete C. Mulhovo S. Molnar J. Ferreira M.-J. U. (2016). Triterpenoids from *Momordica Balsamina*: Reversal of ABCB1-Mediated Multidrug Resistance. Bioorg. Med. Chem. 24, 5061–5067. 10.1016/j.bmc.2016.08.022 27591010

[B116] Ramalhete C. Spengler G. Martins A. Martins M. Viveiros M. Mulhovo S. (2011). Inhibition of Efflux Pumps in Meticillin-Resistant *Staphylococcus aureus* and *Enterococcus faecalis* Resistant Strains by Triterpenoids from Momordica Balsamina. Int. J. Antimicrob. Agents 37, 70–74. 10.1016/j.ijantimicag.2010.09.011 21075604

[B117] Rolnik A. Olas B. (2020). Vegetables from the Cucurbitaceae Family and Their Products: Positive Effect on Human Health. Nutrition 78, 110788. 10.1016/j.nut.2020.110788 32540673

[B118] Roškar R. Lušin T. T. (2012). Analytical Methods for Quantification of Drug Metabolites in Biological Samples. Chromatography–The Most Versatile Method Chem. Anal., 79–126. 10.5772/51676

[B119] Saad D. Y. Soliman M. M. Baiomy A. A. Yassin M. H. El-Sawy H. B. (2017). Effects of Karela (Bitter Melon; *Momordica Charantia*) on Genes of Lipids and Carbohydrates Metabolism in Experimental Hypercholesterolemia: Biochemical, Molecular and Histopathological Study. BMC Complement. Altern. Med. 17, 319. 10.1186/s12906-017-1833-x 28623919 PMC5474009

[B120] Santhosh Kumar J. U. Ramakrishan M. Seethapathy G. S. Krishna V. Uma Shaanker R. Ravikanth G. (2020). DNA Barcoding of *Momordica* Species and Assessment of Adulteration in Momordica Herbal Products, an Anti-diabetic Drug. Plant Gene 22, 100227. 10.1016/j.plgene.2020.100227

[B121] Santos K. K. A. Matias E. F. F. Sobral-Souza C. E. Tintino S. R. Morais-Braga M. F. B. Guedes G. M. M. (2012). Trypanocide, Cytotoxic, and Antifungal Activities ofMomordica Charantia. Pharm. Biol. 50, 162–166. 10.3109/13880209.2011.581672 22235885

[B122] Sathasivam R. Park C. H. Yeo H. J. Park Y. E. Kim J. K. Park S. U. (2021). Analysis of Triterpenoids, Carotenoids, and Phenylpropanoids in the Flowers, Leaves, Roots, and Stems of White Bitter Melon (Cucurbitaceae, *Momordica Charantia*). Trop. J. Pharm. Res. 20 (1), 155–160. 10.4314/tjpr.v20i1.22

[B123] Schrot J. Weng A. Melzig M. (2015). Ribosome-inactivating and Related Proteins. Toxins 7, 1556–1615. 10.3390/toxins7051556 26008228 PMC4448163

[B124] Scorzoni L. Benaducci T. Almeida A. M. F. Silva D. H. S. Bolzani V. d. S. Gianinni M. J. S. M. (2007). The Use of Standard Methodology for Determination of Antifungal Activity of Natural Products against Medical Yeasts *Candida* Sp and *Cryptococcus* Sp. Braz. J. Microbiol. 38, 391–397. 10.1590/s1517-83822007000300001

[B125] Semenya S. S. Potgieter M. J. (2015). Kirkia Wilmsii : A Bapedi Treatment for Hypertension. South Afr. J. Bot. 100, 228–232. 10.1016/j.sajb.2015.05.029

[B126] Sen A. Batra A. (2012). Evaluation of Antimicrobial Activity of Different Solvent Extracts of Medicinal Plant: Melia Azedarach L. Int. J. Curr. Pharm. Res. 4.

[B127] Shah S. Hussain M. Aslam M. Rivera G. (2014). Natural Products; Pharmacological Importance of Family Cucurbitaceae: a Brief Review. Mrmc 14, 694–705. 10.2174/1389557514666140820113055 25138091

[B128] Souda S. George S. Mannathoko N. Goercke I. Chabaesele K. (2018). Antioxidant and Antibacterial Activity of Methanol Extract of *Momordica Balsamina* . IRA-JAS 10, 7–17. 10.21013/jas.v10.n2.p1

[B129] Spengler G. Ramalhete C. Martins M. Martins A. Serly J. Viveiros M. (2009). Evaluation of Cucurbitane-type Triterpenoids from *Momordica Balsamina* on P-Glycoprotein (ABCB1) by Flow Cytometry and Real-Time Fluorometry. Anticancer Res. 29, 3989–3993. 19846941

[B131] Talukdar S. N. Hossain M. N. (2014). Phytochemical, Phytotherapeutical and Pharmacological Study of *Momordica Dioica* . Evid. Based Complement. Alternat Med. 2014, 806082. 10.1155/2014/806082 25197312 PMC4145798

[B132] Tan C. Zuo J. Yi X. Wang P. Luo C. Hu Y. (2015a). Phenolic Constituents from Sarcopyramis Nepalensis and Their α-Glucosidase Inhibitory Activity. Afr. J. Trad. Compl. Alt. Med. 12, 156–160. 10.4314/ajtcam.v12i3.20

[B133] Tan E. S. Abdullah A. Kassim N. K. (2015b). Extraction of Steroidal Glycoside from Small-Typed Bitter Gourd (*Momordica Charanti*a L.). J. Chem. Pharm. Res. 7, 870–878.

[B134] Tan M.-J. Ye J.-M. Turner N. Hohnen-Behrens C. Ke C.-Q. Tang C.-P. (2008). Antidiabetic Activities of Triterpenoids Isolated from Bitter Melon Associated with Activation of the AMPK Pathway. Chem. Biol. 15, 263–273. 10.1016/j.chembiol.2008.01.013 18355726

[B135] Tansey J. Sztalryd C. Hlavin E. Kimmel A. Londos C. (2004). The Central Role of Perilipin a in Lipid Metabolism and Adipocyte Lipolysis. IUBMB Life (International Union Biochem. Mol. Biol. Life) 56, 379–385. 10.1080/15216540400009968 15545214

[B136] Thakur G. Bag M. Sanodiya B. Bhadauriya P. Debnath M. Prasad G. (2009). *Momordica Balsamina*: a Medicinal and Neutraceutical Plant for Health Care Management. Cpb 10, 667–682. 10.2174/138920109789542066 19751180

[B137] Tokhtar V. K. Doan H. G. (2014). Ecological and Biological Features of Tropical Species of the Genus *Momordica* (Cucurbitaceae) Introduced under the Conditions of Belgorod Region (Russia). Russia: Belgorod State National Research University Pobeda Street, 85, Belgorod, 308015.

[B138] Trakoon-Osot W. Sotanaphun U. Phanachet P. Porasuphatana S. Udomsubpayakul U. Komindr S. (2013). Pilot Study: Hypoglycemic and Antiglycation Activities of Bitter Melon (*Momordica Charantia* L.) in Type 2 Diabetic Patients. J. Pharm. Res. 6, 859–864. 10.1016/j.jopr.2013.08.007

[B139] Venugopal D. Dhanasekaran S. (2021). Bitter Gourd (*Momordica Charanti*a) as an Emerging Therapeutic Agent: Modulating Metabolic Regulation and Cell Signaling Cascade. Stud. Nat. Prod. Chem. 67, 221–268. 10.1016/B978-0-12-819483-6.00007-2

[B140] Waako P. J. Gumede B. Smith P. Folb P. I. (2005). The In Vitro and In Vivo Antimalarial Activity of C*ardiospermum Halicacabum* L. And *Momordica Foetid*a Schumch. Et Thonn. J. Ethnopharmacology 99, 137–143. 10.1016/j.jep.2005.02.017 15848033

[B141] Wang S. Zheng Y. Xiang F. Li S. Yang G. (2016). Antifungal Activity of Momordica Charantia Seed Extracts toward the Pathogenic Fungus *Fusarium Solan*i L. J. Food Drug Anal. 24, 881–887. 10.1016/j.jfda.2016.03.006 28911628 PMC9337286

[B142] Wang X. Sun W. Cao J. Qu H. Bi X. Zhao Y. (2012). Structures of New Triterpenoids and Cytotoxicity Activities of the Isolated Major Compounds from the Fruit of *Momordica Charantia* L. J. Agric. Food Chem. 60, 3927–3933. 10.1021/jf204208y 22369241

[B143] Wardani B. P. N. Rifa’i M. Rahayu S. (2021). Bitter Melon (Momordica charantia L.) and Star fruit (Averrhoa bilimbi L.) on Proinflammatory Cytokines Produced by Hyperglycemic Mice Model. J. Exp. Life Sci. 10 (2), 89–93.

[B144] Wardhani T. Abadi A. L. Himawan T. Am A. (2015). Composition of Water Extract from Wild Bitter Gourd (*Momordica Charantia* L.) Fruit for Application as Antifeedant and Mortality Test on Armyworm Larvae (Spodoptera Litura Fab.). J. Biol. Life Sci. 6. 10.5296/jbls.v6i2.8084

[B145] Wikaningtyas P. Sukandar E. Y. (2016). The Antibacterial Activity of Selected Plants towards Resistant Bacteria Isolated from Clinical Specimens. Asian Pac. J. Trop. Biomed. 6, 16–19. 10.1016/j.apjtb.2015.08.003

[B146] Yoshime L. T. De Melo I. L. P. Sattler J. a. G. De Carvalho E. B. T. Mancini-Filho J. (2016). Bitter Gourd (*Momordica Charantia* L.) Seed Oil as a Naturally Rich Source of Bioactive Compounds for Nutraceutical Purposes. Nutrire 41, 12. 10.1186/s41110-016-0013-y

[B147] Yue J. Sun Y. Xu J. Zhang X. Zhao Y. (2020). Four New Cucurbitane-type Triterpenes from Momordica Charantia L. With Their Cytotoxic Activities and Protective Effects on H2O2-Damaged Pancreatic Cells. J. Nat. Med. 74, 34–40. 10.1007/s11418-019-01336-1 31256310

[B148] Yuwai K. E. Rao K. S. Kaluwin C. Jones G. P. Rivett D. E. (1991). Chemical Composition of *Momordica Charantia* L. Fruits. J. Agric. Food Chem. 39, 1762–1763. 10.1021/jf00010a013

